# Barley transcriptome analyses upon interaction with different aphid species identify thionins contributing to resistance

**DOI:** 10.1111/pce.12979

**Published:** 2017-07-18

**Authors:** C.M. Escudero‐Martinez, J.A. Morris, P.E. Hedley, J.I.B. Bos

**Affiliations:** ^1^ Cell and Molecular Sciences The James Hutton Institute Dundee DD2 5DA UK; ^2^ Division of Plant Sciences, School of Life Sciences University of Dundee Dundee DD2 5DA UK

**Keywords:** herbivore, host range, plant defence, transcriptomics

## Abstract

Aphids are phloem‐feeding insects that cause yield loss on a wide range of crops, including cereals such as barley. Whilst most aphid species are limited to one or few host species, some are able to reproduce on many plants belonging to different families. Interestingly, aphid probing behaviour can be observed on both host and non‐host species, indicating that interactions take place at the molecular level that may impact host range. Here, we aimed to gain insight into the interaction of barley with aphid species differing in their ability to infest this crop by analysing transcriptional responses. Firstly, we determined colonization efficiency, settlement and probing behaviour for the aphid species Rhopalosiphum padi, Myzus persicae and *Myzus cerasi*, which defined host, poor‐host and non‐host interactions, respectively. Analyses of barley transcriptional responses revealed gene sets differentially regulated upon the different barley–aphid interactions and showed that the poor‐host interaction with M. persicae resulted in the strongest regulation of genes. Interestingly, we identified several thionin genes strongly up‐regulated upon interaction with M. persicae, and to a lesser extent upon R. padi interaction. Ectopic expression of two of these genes in *Nicotiana benthamiana* reduced host susceptibility to M. persicae, indicating that thionins contribute to defences against aphids.

## Abstract 2

Non‐host and poor‐host resistance to aphids is poorly understood and is key for development of novel crop resistances to insect pests. Here, we used barley as a monocot model crop plant to study the interaction with aphid species that differ in their ability to infest and analysed barley transcriptional responses during interactions. Our work provides insights into how barley responds to different types of aphid interactions. Importantly, we identified barley genes contributing to plant defences against aphids.

## Introduction

Aphids are phloem‐feeding insects that cause substantial yield loss on a wide range of crop plants, including monocots and dicots. Most aphid species have a narrow host range, limited to one or few plant families. However, some aphids are able to infest a wide range of plant species, including many important agricultural crops. One example is Myzus persicae (green peach aphid), which can infest plants in over 40 families, including solanaceous crops such as potato and tomato, cucurbits, legumes and ornamentals (Blackman & Eastop 2000). In contrast, closely related *Myzus cerasi* (black cherry aphid) is only able to infest a small number of herbaceous plants. Other species, like Rhopalosiphum padi, are highly specialized to infest grasses. Host selection by aphids involves a complex set of plant cues and signals and, most likely, molecular interactions that take place between plant and aphid upon probing and feeding (as reviewed by Powell *et al*. 2006; Jaouannet *et al*. 2015).

Upon landing on the leaf surface, aphids may detect plant cues and structures that impact their behaviour. During the interaction with a compatible host plant, aphids will use their specialized mouthparts, or stylets, to feed from the phloem. Once the stylets penetrate the leaf epidermal cells, they follow a mainly extracellular pathway to reach the phloem (Tjallingii 1995). Furthermore, the stylets briefly puncture individual cells along the stylet pathway to potentially detect plant cues. Saliva is secreted during both probing and feeding, which contains sets of proteins and small molecules, called effectors, that promote aphid virulence in compatible interactions (Will *et al*. 2007; Hogenhout & Bos, 2011; Elzinga & Jander 2013). Interestingly, aphid probing takes place not only on host plant species but also on non‐hosts, suggesting that recognition and activation of resistance may be dependent on the perception of molecules in aphid saliva (Powell *et al*. 2006). Indeed, aphid saliva exhibits elicitor activity and can trigger responses similar to pathogen‐associated molecular pattern‐triggered immunity (PTI) in plant–pathogen interactions (De Vos & Jander 2009; Chaudhary *et al*. 2014). Although these studies all focused on Arabidopsis as a model host, elicitor activity has also been reported in whole extracts of *Diuraphis* *noxia* (Russian wheat aphid) on a resistant wheat (Triticum aestivum L.) genotype (Lapitan *et al*. 2007). These observations imply that plants recognize aphid saliva components to trigger defences. Whether such recognition events are indeed determinants of aphid host range remains to be investigated.

Aphid infestations can cause significant yield losses in cereals worldwide. One of the major aphid pests of cereals is Rhopalosiphum padi, which infests wheat, barley and oats. This aphid not only causes direct feeding damage resulting in yield losses up to 50% (Kieckhefer & Kantack 1986; Papp & Mesterhazy 1993) but also transmits *barley yellow dwarf virus*, which infects cereals and grasses, and causes reduced growth and leaf yellowing (Oswald & Houston 1953; Riedell *et al*. 1999). Barley (Hordeum vulgare L.) is a major economical cereal crop, but also a model monocot plant for molecular biology and genetics research. Although no resistant commercial barley cultivars are available against R. padi, partial resistant genotypes were previously identified (Delp *et al*. 2009). Gene expression analyses of susceptible versus partially resistant barley genotypes upon R. *padi* interaction identified a few genes specifically induced in resistant plants (Delp *et al*. 2009). These included genes encoding a Ser/Thr kinase, a calcium‐binding EF‐hand protein (BCI4), a proteinase inhibitor and lipoxygenase 2 (LOX2). A number of genes were more highly expressed in resistant versus susceptible genotypes in the absence of aphid infestation, including genes encoding thionins (Delp *et al*. 2009; Mehrabi *et al*. 2014).

Although oxidative stress responses play an important role in several plant–aphid interactions, R. *padi* does not elicit peroxidase activity or consistently activate peroxidase genes in barley (Ni *et al*. 2001; Delp *et al*. 2009). Moreover, only limited callose deposition is triggered upon interaction of barley with R. *padi* as compared with other cereal aphids, indicating that plant defence responses may differ depending on both host and aphid species (Saheed *et al*. 2007; Saheed *et al*. 2008).

The broad host range aphid M. *persicae* is not considered a pest of barley. However, this aphid has been reported on wheat and barley under field conditions (Halbert & Pike 1985). Despite this, M. *persicae* performed poorly on barley under controlled growth chamber conditions and showed low levels of colonization and limited ingestion of phloem sap on barley when compared with wheat (Davis & Radcliffe 2008). Based on these observations, barley can be considered a poor‐host species of M. *persicae*. Here, we were interested to investigate how barley responds to different aphid species, including R. *padi* and M. *persicae*, to gain insight into why this plant is a suitable host for specific aphid species. Specifically, we aimed to gain insight into why M. *persicae* performs poorly on barley despite an exceptionally broad host range, which includes species within the *Poaceae*.

We previously dissected plant transcriptional responses to aphids during host, poor‐host and non‐host interactions in Arabidopsis (Jaouannet *et al*. 2015). This revealed several genes specifically involved in either host susceptibility to M. *persicae* or non‐host resistance to R. *padi* (bird cherry‐oat aphid). Here, we used a combination of aphid interaction assays and barley transcriptomics to assess how a monocot crop species responds to different types of aphid interactions and to identify barley genes that may contribute to defences against aphids.

## Materials and Methods

### Aphid cultures

Aphids used for all the experiments were maintained under controlled conditions in growth chambers (18 °C, 16 h of light) and contained in cages. R. *padi* was raised on H. vulgare L. cv. Optic, M. *persicae* (genotype O) was reared on Brassica napus (oilseed rape) and M. cerasi was raised on Barbarea verna (land cress). R. *padi* and M. *persicae* were kindly provided by Dr B. Fenton, and M. *cerasi* was collected from cherry trees in Dundee (UK).

### Barley colonization assays

Seven‐day‐old barley plants of different cultivars (Golden Promise, Optic and Morex) were infested with each of two first‐instar nymphs of the species R. *padi*, M. *persicae* and M. *cerasi*. We performed colonization experiments in three biological replicates with seven individually bagged plants for each aphid species per replicate. The total number of aphids was monitored at 8, 14 and 20 d after placing nymphs on the plants. To compare M. *persicae* colonization of barley versus oilseed rape in parallel, three 4‐week‐old oil seed rape plants and three 7‐day‐old barley cv. Golden Promise plants were each challenged with two first‐instar nymphs. The total number of aphids was recorded at 7 and 14 d after challenge (DAC). Statistical analysis for the aforementioned experiments was performed using the Shapiro–Wilk test for normality and a one‐way non‐parametric test (Mann–Whitney) in Genstat. We measured the length and width of M. *persicae* aphids reared on oil seed rape and the three different barley cultivars 7 d after aphid challenge. Each plant was challenged with 10 first‐instar nymphs. The length and width (mm) of aphid were measured in images taken from a set distance with the software imagej (Schneider *et al*. 2012). Statistical analyses were performed using anova, with a single factor for the parameters' length and width. All experiments were performed in growth chambers (18 °C, 16 h of light).

### Aphid settlement assays

Seven‐day‐old barley plants (cv. Optic) were used to assess R. *padi*, M. *persicae* and M. *cerasi* settlement on barley leaves. Four clip cages per aphid species were prepared containing 10 aphids of each species per clip cage. At time 0, the cages, containing the aphids, were placed on the barley leaves. At intervals of 15 min, the clip cages were opened and the number of aphids settled on the barley leaf was counted. The experiment was performed as three biological replicates. The results were analysed by analysis of variance (anova) with Fisher's protected least significant difference *post hoc* test per timepoint.

### Aphid probing assays

Barley leaf samples (1 cm^2^) were placed on 24‐well plates containing 1% water agar. Subsequently, four age‐synchronized adult aphids from the species R. *padi*, M. *persicae* or M. *cerasi* were each placed on the leaf samples (cv. Golden promise, Optic and Morex). Six leaf samples were used per aphid species, and three independent replicates were set up. Plates with aphids were kept in short‐day chambers at 22 °C. After 2 d, the leaf samples were stained in an acid fuchsine solution (Urbanska 2010) and analysed under a light microscope for the presence of stylet pathways. Stylet paths were counted for each 1 cm^2^ leaf sections challenged with aphids and classified as long pathways or short probes. Differences between path and probes were assessed by anova with Fisher's protected least significant difference *post hoc* test (*P* > 0.05). We used a similar set‐up to visualize callose with aniline blue staining. We removed the chlorophyll from the barley leaves (cv. Optic) using (1:3) acetic acid:ethanol over 8 h with two changes in a constant shaker. Next, samples were incubated for 24 h in a solution of 0.05% aniline blue (protocol adapted from Daudi *et al*. 2012). Samples were analysed for the presence of cells with callose deposition under a confocal microscope Zeiss LSM 510 (Jena, Germany) using a Zeiss ×20 lens and a green excitation filter (wavelength 516 nm).

### Barley transcriptome analyses

Barley plants (cv. Optic) were pre‐germinated in Petri dishes covered with wet filter paper for 3 d in the dark. Germinating seeds were then moved to soil and grown under controlled conditions (short day, at 22 °C, 70% humidity and 125 μmol photons m^−2^s^−1^). One week later, the plants were challenged with 15 mixed‐age aphids enclosed in clip cages. As a control, we also placed clip cages without aphids on barley plants. Leaf tissues enclosed within the clip cages were collected after 3 and 24 h from both aphid‐challenged and control treatments. Samples from the same treatment (six samples) were pooled together in a Falcon tube submerged in liquid nitrogen. We performed this experiment as three biological replicates. Individual replicates were set up at the same time of day to take into account any effects of the plant circadian cycle. The experiment was started at 0900 h, with the 3 h samples being collected from 1200 h and the 24 h samples being collected from 0900 h the next day. RNA was extracted using the Qiagen RNeasy Plant Mini Kit® following the manufacturer's protocol (Qiagen, Hilden, Germany). RNA quality was assessed using the Agilent 2100 Bioanalyzer prior to microarray processing (Agilent Technologies, Santa Clara, CA, USA). RNA from each of three replicates was hybridized to a custom‐design Agilent barley 60K microarray (Comadira *et al*. 2015). The microarray experimental design and dataset can be accessed at ArrayExpress (https://www.ebi.ac.uk/arrayexpress; accession # E‐MTAB‐5133).

Recommended total RNA labelling (100 ng each) and hybridization were used throughout (Agilent One‐Color Microarray‐Based Gene Expression Analysis: Low Input Quick Amp Labeling version 6.5). Data were extracted from each microarray using feature extraction software (version 10.7.3.1; Agilent Technologies, Santa Clara, CA, USA) with default settings, and subsequently, data were imported into genespring (version 7.3; Agilent Technologies, Santa Clara, CA, USA) software for preprocessing and analyses. Default one‐colour normalization was performed, and probes were filtered on flags to remove inconsistent data. To identify genes differentially expressed between aphid‐challenged versus unchallenged barley samples at the 3 and 24 h timepoints, a paired Student's *t*‐test (*P*‐value ≤0.05 with Benjamini–Hochberg correction) was performed. Data were visualized using line graphs, box whisker plots, tree–heat maps for hierarchical clustering and Venn diagrams for gene list comparisons. MapMan functional BIN classification was performed using Wilcoxon rank sum test (cut‐off *P* ≤ 0.05 and fold change ≥2) (Thimm *et al*. 2004).

We assessed variation between the 3 and 24 h control samples to determine the impact of potential diurnal effects or different exposure time to clip cages on hierarchical clustering. By comparing the two controls, we identified 331 genes differentially expressed. Of these, 120 were also identified in our set of 974 differentially expressed genes across barley–aphid interactions. Removing these 120 genes from the hierarchical clustering approach did not affect the main groups identified across barley aphid interactions as shown in Fig. 3 (Supporting Information Fig. S1 and Table S11).

### Gene ontology enrichment analyses

We used Biomaps software available on the Virtual Plant web platform, version 1.3 (http://virtualplant.bio.nyu.edu/cgi-bin/vpweb) (Katari *et al*. 2010), to analyse gene ontologies (GOs) using Arabidopsis thaliana Columbia tair10 genome (28 775 genes) as a reference genome. Gene enrichment analysis of the set of genes was performed in Biomaps. The different gene sets were interrogated with the available options: GO biological process, GO molecular function and GO cellular compartments (TAIR/TIGR), AraCyc pathways (v11.5) from PlantCyc, functional classification by the Munich Information Center for Protein Sequences and KEGG pathways. The GO was calculated with Fisher's exact test *P* ≤ 0.05 (false discovery rate correction).

### Quantitative RT‐PCR to assess gene expression changes

Validation of the microarray data and analyses of gene expression changes across selected barley cultivars was performed by qRT‐PCR using the Universal Probe Library (Roche Diagnostics, Basel, Switzerland). To validate the expression of selected genes, we pooled the RNA of the three barley cv. Optic biological replicates used for microarray hybridization. For expression analyses of barley thionins at 72 h post infestation, we used three biological replicates of barley cultivar Optic challenged with aphids for 72 h using clip cages as described for the microarray experiment. To assess expression of selected genes across cultivars, we used three biological replicates of barley cultivars Morex and Golden Promise challenged with aphids for 24 h using clip cages as described for the microarray experiment with cultivar Optic. DNAse‐treated RNA (Ambion TURBO DNA‐free DNase treatment; ThermoFisher Scientific, Waltham, MA, USA) was converted into cDNA with SuperScript III Reverse Transcriptase (ThermoFisher Scientific (Invitrogen), Waltham, MA, USA) using random primers. Databases used for primer design were a local database containing predicted barley cv. Morex genes, NCBI, Ensembl Plants H. vulgare and the Plant Genome and Systems Biology barley genome database. Primers and probes were designed using the Universal Probe Library Roche website and are listed in Supporting Information Table S10. Primers and probes were first tested for efficiency (85–105%). Reactions were prepared using 25 μL of total volume, 12.5 μL of FastStart TaqMan Probe Master Mix (containing ROX reference dye) and 0.25 μL of gene specific primers (0.2 mM) and probes (0.1 mM). A StepOne thermocycler (ThermoFisher Scientific (Applied Biosystems), Waltham, MA, USA) was run as follows: 10 min of denaturation at 95 °C, followed by 40 cycles of 15 s at 94 °C and 60 s at 60 °C. Relative gene expression was calculated with the method ΔΔCt (Delta Cycle threshold) with primer efficiency taken into consideration. Every sample was run as three technical replicates. Cycle threshold values were normalized with three reference genes, *actin‐2* (*MLOC_78511.2*), *ubiquitin* (*AK248472.1*) and *pentatricopeptide* (*AK373147*/*MLOC_80089.1*). Expression of these reference genes was unaffected in our microarray experiment (data not shown).

### Ectopic expression of thionins followed by aphid performance assays

Two different barley thionin genes (*AK252675.1*/*MLOC_46400.1* and *AK359149*/*MLOC_34881.1*) were selected for cloning and aphid performance assays. Coding sequences were cloned into destination vectors pB7WG2 to allow ubiquitous overexpression under the 35S promoter. *Agrobacterium tumefaciens* strain GV3101 carrying the thionin constructs or the empty vector was infiltrated into *Nicotiana benthamiana* leaves at a relative OD_600_ = 0.1. Twelve infiltration sites were used for each construct (two per plant, a total of 6 plants per construct). One day after infiltration, two adult M. *persicae* aphids were placed at the underside of the infiltrated leaf areas and the area was enclosed with a clip cage. One day later, the adult aphids and all except three nymphs were removed from the leaf area. Aphids were moved to freshly infiltrated plants 7 d after initial agroinfiltration. Total nymph numbers were counted 14 d after the start of the experiment. Three independent biological replicates were performed. Differences between thionin‐expressing leaves and the vector control were assessed by one‐way anova and *post hoc* Fisher's test (*P* ≤ 0.01).

To assess activation of *N. benthamiana* defence genes *PAD4*, *PR‐1*, *TP1* and *PR‐4*, by barley thionins we agroinfiltrated the leaves of three plants per biological replicate and collected leaf samples for RNA extraction with the Qiagen RNeasy Plant Mini Kit® (Qiagen, Hilden, Germany). Quantitative RT‐PCR was performed as previously described by Rodriguez *et al*. 2014.

## Results

### Differences in barley colonization by the aphid species Myzus *persicae*, *Myzus cerasi* and Rhopalosiphum padi

**Figure 1:**
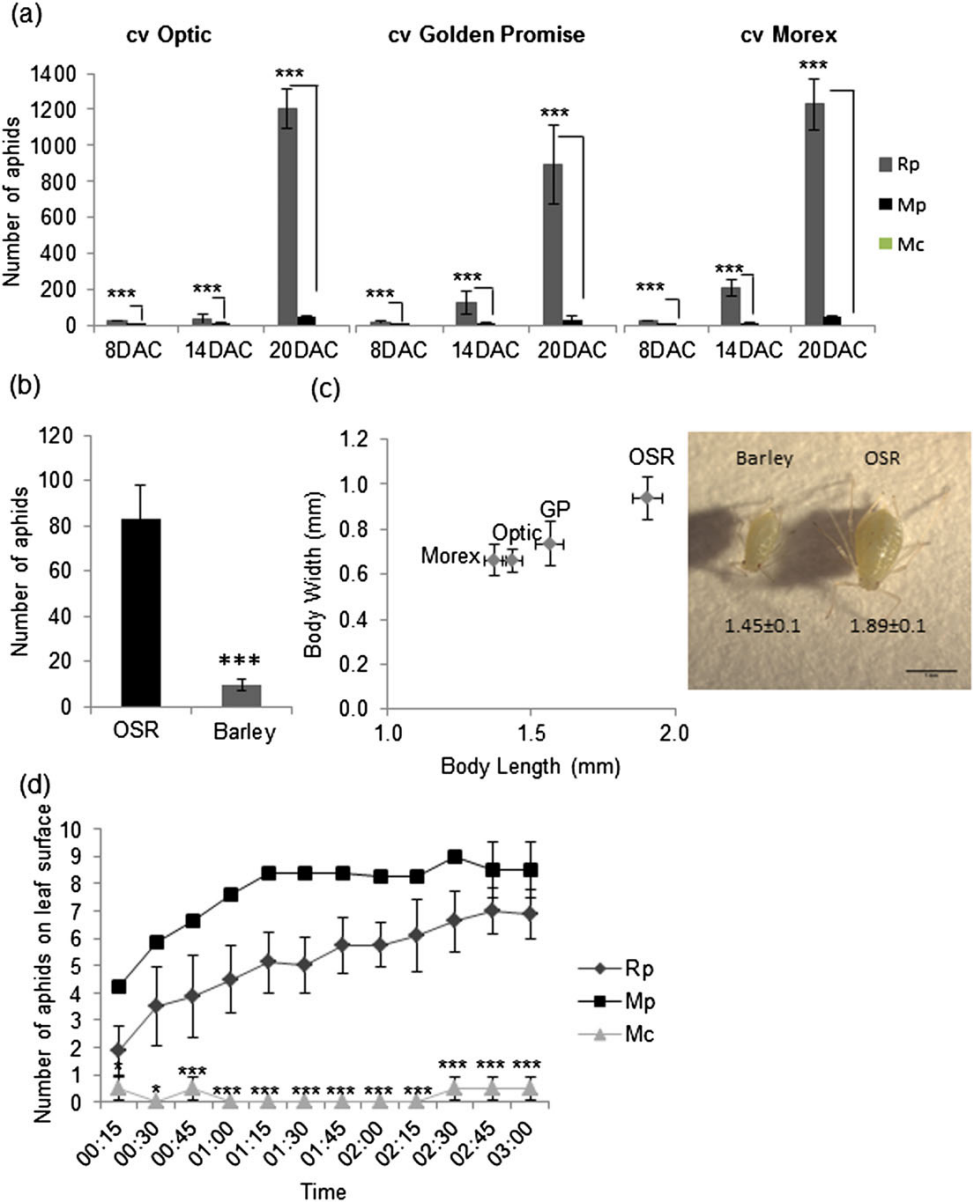
Barley colonization by aphid species Rhopalosiphum padi (Rp), Myzus persicae (Mp) and *Myzus cerasi* (Mc). (a) Number of aphids counted on different barley cultivars [Optic, Golden Promise (GP) and Morex] 8, 14 and 20 d after challenge (DAC) of individual plants with two adult aphids. Error bars show the standard deviation. Triple asterisks indicate statistically significant differences, using a one‐way non‐parametric test Mann–Whitney (*P* ≤ 0.001), between the numbers of Rp and Mp aphids counted. Mc was unable to survive on barley. Seven plants per treatment and replicate were used, and three independent biological replicates were performed. (b) Number of Mp aphids counted on host plant oil seed rape (OSR) and barley cv. GP 14 d after placing two adult aphids on individual plants in parallel. Error bars show the standard deviation. The asterisk indicates significant differences (*P* ≤ 0.01) using a Mann–Whitney test. Three plants per treatment and replicate were used, and three independent biological replicates were performed. (c) Plot of the Mp body length and width when raised on OSR and on barley cultivars GP, Optic and Morex. Error bars indicate standard error. Differences were assessed by one‐way analysis of variance. The image on the right shows a representative individual aphid taken from the population raised on OSR and barley (cv. GP) and was taken 7 d after placing first‐instar nymphs on the different plant species. Ten first‐instar nymphs per treatment were used in each replicate, and three independent biological replicates were performed. (d) Number of aphids counted on barley leaves (cv. Optic) during the first 3 h after placing 10 aphids in clip cages to the underside of leaves. Four plants (1 clip cage per plant) were used per treatment per replicate, and three independent biological replicates were performed. Error bars indicate standard deviation of three independent replicates. Asterisks indicate significant differences in anova with Fisher's protected least significant difference *post hoc* test per timepoint (single asterisk *P* > 0.05, triple asterisk *P* > 0.01).

Although M. *persicae* and M. *cerasi* have not been reported to cause significant infestations on barley, M. *persicae* has been found on barley plants in a field setting, suggesting that this aphid may be able to at least survive on this crop (Halbert & Pike 1985). We were interested to determine whether, and to what extent, both these aphids were able to colonize barley under controlled glasshouse conditions. Therefore, we set up aphid infestation assays of three barley cultivars, Golden Promise, Optic and Morex, with M. *persicae* and M. *cerasi*, as well as R. *padi*, a major aphid pest of barley. Seven‐day‐old plants were challenged each with 2 nymphs per plant, and the numbers of aphids were counted at 8, 14 and 20 DAC (Fig. 1a). Whilst the R. *padi* population reached on average between 800 and 1200 aphids across the cultivars, the M. *persicae* population only reached on average between 30 and 45 aphids at 20 DAC (Fig. 1a). No living aphids were found for M. *cerasi* at 8 DAC, indicating that this species was unable to survive on barley.

Because we found that M. *persicae* was able to reproduce on barley, we were interested to compare how the level of reproduction on barley compared with that on the well‐documented host plant oilseed rape (B. napus). We performed colonization experiments of barley and oil seed rape in parallel for M. *persicae* and infested 7‐day‐old barley and 4‐week‐old oil seed rape plants each with 2 synchronized adult aphids per plant. The total populations were counted after 14 d. Whilst we counted over 80 aphids on oil seed rape, we only found around 10 aphids on barley (cultivar Golden Promise), indicating an eightfold difference in population size (Fig. 1b). During these experiments, we noted that the M. *persicae* adults reared on barley were smaller in size than those on oil seed rape. To confirm this, we measured aphid body length and width of M. *persicae* reared on barley versus oil seed rape. We allowed six nymphs to feed on barley or oilseed rape plants for 7 d and measured body length and width. Aphids feeding on barley were significantly smaller than those feeding on oil seed rape (one‐way anova, *P* ≤ 0.01) (Fig. 1c). No significant differences in aphid size were found between different barley cultivars (Fig. 1c). Although M. *persicae* was able to survive and reproduce on barley, this species performs poorly on this crop plant compared with R. *padi*. Based on our colonization data, we defined M. *cerasi*–barley as a non‐host interaction and M. *persicae*–barley as a poor‐host interaction in our follow‐up work detailed in the succeeding texts.

In our colonization experiments, we noticed a smaller number of aphids remained on the leaf surface for M. *cerasi* than the other species after placing them on barley leaves. This observation led us to further investigate whether the aphid species settled differently on barley in a no‐choice experiment. We placed 10 adult aphids for each species in a clip cage, which was attached to the lower side of the leaf surface, allowing aphids to either stay in the clip cage or move onto the plant. Aphid numbers on the leaf surface were counted at 15 min intervals for 3 h. Whilst both R. *padi* and M. *persicae* moved from the clip cages to the leaf surface, with between seven and nine aphids counted after 3 h, only few aphids (between one and three) were found on the leaf surface in the case of M. *cerasi* (Fig. 1d). This, together with the inability of M. *cerasi* to survive and reproduce on barley, shows barley is a non‐host of this aphid species.

### The aphid species Myzus *persicae*, *Myzus cerasi* and Rhopalosiphum padi produce different stylet pathways when probing barley leaves and activate callose deposition

**Figure 2:**
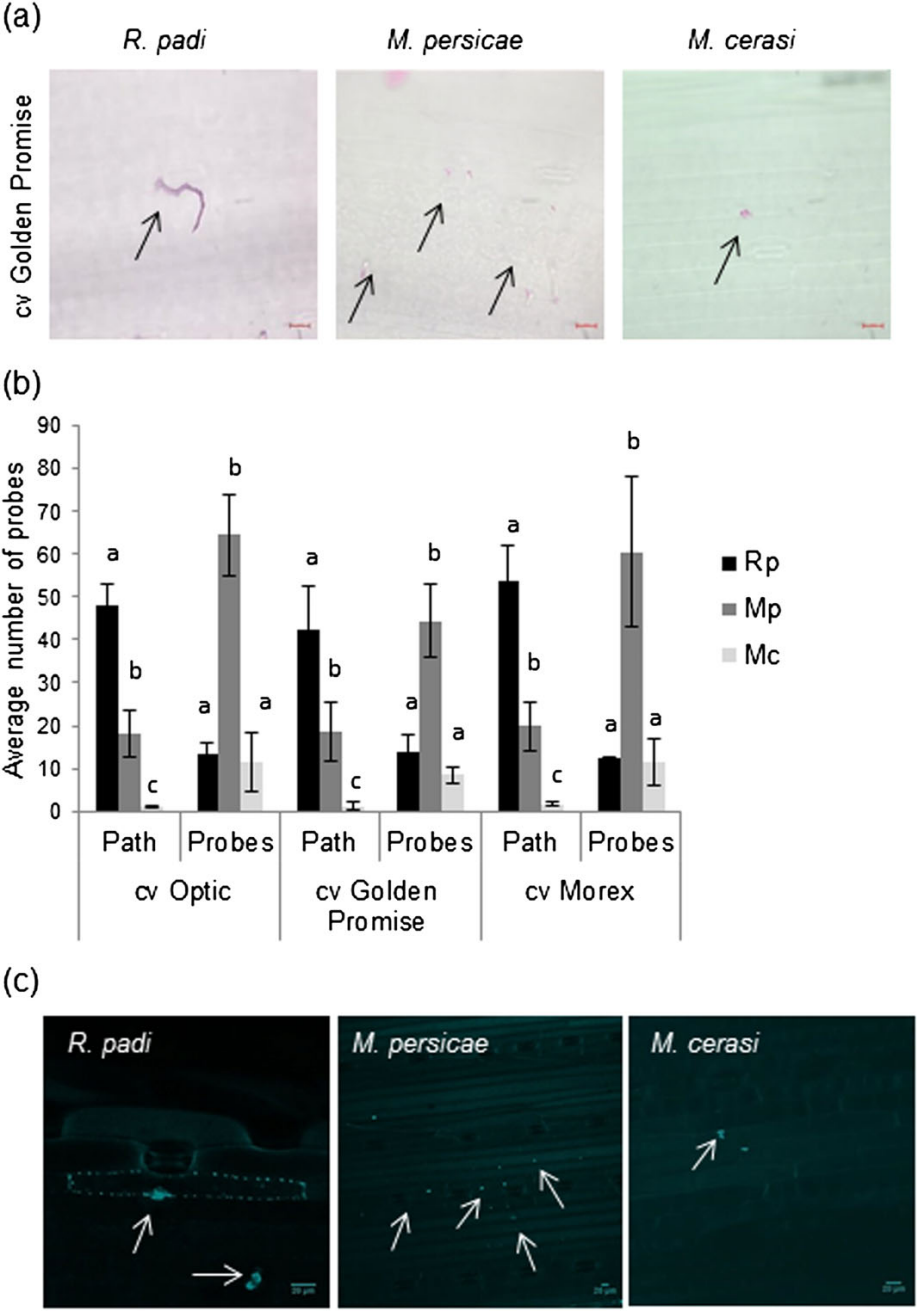
Probing of barley leaf tissue by aphid species *Rhopalosiphum padi* (Rp), *Myzus persicae* (Mp) and *Myzus cerasi* (Mc). (a) Images showing aphid probes and stylet pathways in barley cv. Golden Promise 2 d after aphid challenge visualized by staining with acid fuchsine. Images were taken with a light microscope. The probes and stylet pathways are indicated by arrows. Scale bars are 20 μm. (b) Graph showing numbers of brief probes and stylet pathways as observed in Fig 1a for the different barley–aphid interactions. Four adult aphids per species were placed in 1 cm^2^ leaf sample, and after 2 d, the number of stylet paths or short probes was counted. Six leaf samples were used per aphid species per replicate, and three independent biological replicates were performed. Error bars indicate standard deviation. Different letters indicate significant differences in analysis of variance with Fisher's protected least significant difference *post hoc* test (*P* > 0.05). (c) Representative images showing callose deposition upon aphid probing visualized using aniline blue staining 2 d after aphid challenge. The sites of callose deposition after aphid stylet penetration are indicated by an arrow. Samples were visualized under the confocal microscope using a green filter (wavelength 516 nm). Six leaf samples were used per aphid species per replicate, and the experiment was performed in three independent biological replicates. Scale bars are 20 μm. Additional images are shown in Supporting Information Fig. S3.

Aphid probing has been reported to take place during both host and non‐host interactions and is essential for the delivery of saliva inside plant cells and the apoplastic space (Mclean & Kinsey 1968; Wiktelius 1982). We investigated whether the different aphid species included in our study were all probing barley leaves and how stylet pathways compared among the different interactions. To do this, we made use of an acid fuchsine stain, which is commonly used to visualize aphid stylet pathways, in combination with light microscopy. We challenged barley leaves with R. *padi* (host interaction), M. *persicae* (poor‐host interaction) or M. *cerasi* (non‐host interaction) and collected leaf samples for staining 2 d later. For R. *padi*, we mainly observed long highly branched stylet pathways, whereas for M. *cerasi*, which was unable to survive on barley, we observed a small number of short probes, visible as pink dots (Fig. 2a). For M. *persicae*, we observed a large number of short probes, visible as pink dots, but also some stylet pathways (Fig. 2a and Supporting Information Fig. S2). We then quantified the number of pathways and short probes (dots without pathway) in a 1‐cm^2^‐size leaf area of three different barley cultivars (Morex, Optic and Golden Promise). This confirmed that during the host interaction with R. *padi*, long and branched stylet pathways were most abundant, with between 40 and 50 pathways per leaf area and less than 20 short probes (Fig. 2b). During the poor‐host interactions with M. *persicae,* we mostly detected short probes, ranging from 40 to 60 probes per leaf area and around 20 stylet pathways (Fig. 2b). During the non‐host interaction with M. *cerasi*, we observed a much lower number of short probes (around 10) per leaf area than the poor‐host interaction with M. *persicae*, and only few stylet pathways. We observed similar results across the three barley cultivars (Fig. 2b). Our data show that although M. *cerasi* and M. *persicae* do not or poorly infest barley, these aphids probe the barley leaf tissue, indicating that signals can be exchanged at the plant–aphid stylet interface. In addition, clear differences in stylet pathway formation were observed, which may reflect the ability of the aphids to successfully feed from the phloem and establish populations.

During compatible host barley–aphid interactions, the production of callose depositions has been reported (Saheed *et al*. 2009). Therefore, we assessed whether barley responds to M. *persicae* (poor‐host interaction), M. *cerasi* (non‐host interaction) and R. *padi* (host interaction), in a similar way with regard to callose deposition. We visualized callose using aniline blue staining on barley epidermal cells from leaves 2 d after infestation with five adult aphids. We observed a strong callose accumulation at the site of the stylet penetration in the epidermal cells for all three interactions (Fig. 2c and Supporting Information Fig. S3). For R. *padi*, we usually observed one stylet pathway or probe per cell and we also noted callose depositions at the cell wall of punctured cells where we detected a stylet pathway (Fig. 2c). For M. *persicae*, we noted multiple sites of callose deposition per cell, which most likely reflects multiple probing sites per cell (Fig. 2c). These results are consistent with our observations that M. *persicae* shows increased probing compared with R. *padi*. Occasionally, cell wall depositions could be observed during barley–M. *persicae* interactions. For M. *cerasi*, we only occasionally detected callose depositions, which again likely reflects limited probing consistent with the results obtained using fuchsine staining and light microscopy (Fig. 2c).

### The barley transcriptome responds most strongly to interaction with *Myzus persicae* compared with the interactions with *Rhopalosiphum padi* and *Myzus cerasi*

**Figure 3:**
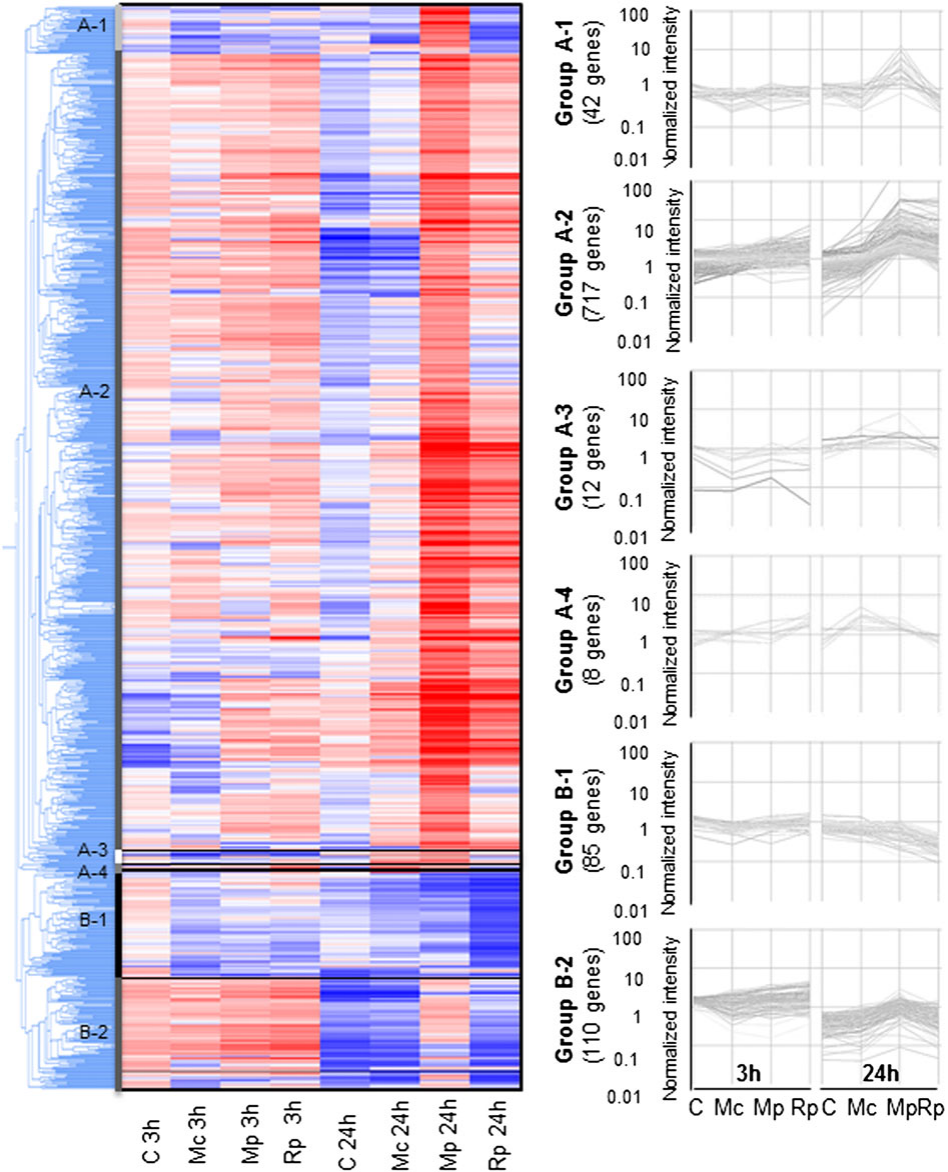
Hierarchical clustering of differentially expressed barley genes among the different aphid treatments and controls. Significantly changing genes were identified using paired *t*‐test comparisons (Benjamini–Hochberg correction, *P*‐value ≤ 0.05) between the aphid treatment and the corresponding no‐aphid control for each timepoint. The tree represents all 974 genes differentially expressed in comparisons to the no‐aphid control. Hierarchical gene tree cluster analysis of the 974 genes identified two main clusters (A and B). Cluster A was divided into four sub‐clusters, and cluster B comprised two sub‐clusters. Blue colour indicates low expression level, and red colour indicates high expression level. No‐aphid control (C), *Rhopalosiphum padi* (Rp), *Myzus persicae* (Mp) and *Myzus cerasi* (Mc) treatments are indicated.

We previously compared Arabidopsis host and poor‐host and non‐host responses to aphids, which identified genes differentially expressed during specific interactions (Jaouannet *et al*. 2015). Here, we aimed to perform a similar comparison using barley as a model monocot crop species. We challenged 7‐day‐old barley plants (cv. Optic) each with 15 adults of M. *persicae*, M. *cerasi* and R. *padi* aphids or no aphids and collected leaf material 3 and 24 h later. RNA was extracted and prepared for hybridization with a custom Agilent 60K barley microarray. We identified 974 genes that were significantly differentially expressed (*P*‐value ≤0.05) in at least one of the aphid treatments compared with the no‐aphid control (Supporting Information Table S1). Hierarchical gene tree cluster analysis of the differentially expressed genes revealed two main clusters within this set of genes based on their expression profiles (Fig. 3). Cluster A comprised 779 genes and cluster B 195 genes. Within these two main clusters, we identified sub‐clusters that behave differently across the treatments within the main cluster (Fig. 3 and Supporting Information Table S2). Two sub‐clusters, A‐2 and B‐2, showed significant over‐representation of gene functional categories based on GO annotation. Sub‐cluster A‐2 comprised 717 genes, which were predominantly up‐regulated during the interactions with R. *padi* and M. *persicae* at the 24 h timepoint, and cluster B‐2, 110 genes specifically up‐regulated at the 24 h timepoint during the M. *persicae* interaction (Fig. 3 and Supporting Information Table S2). Both these clusters showed over‐representation of genes predicted to be involved in a range of metabolic processes with functions in catalytic activity (GO:0003824) (Supporting Information Tables S2 and S3).

### Differential barley transcriptome responses specific to the interaction with *Rhopalosiphum padi* or *Myzus persicae*

**Figure 4:**
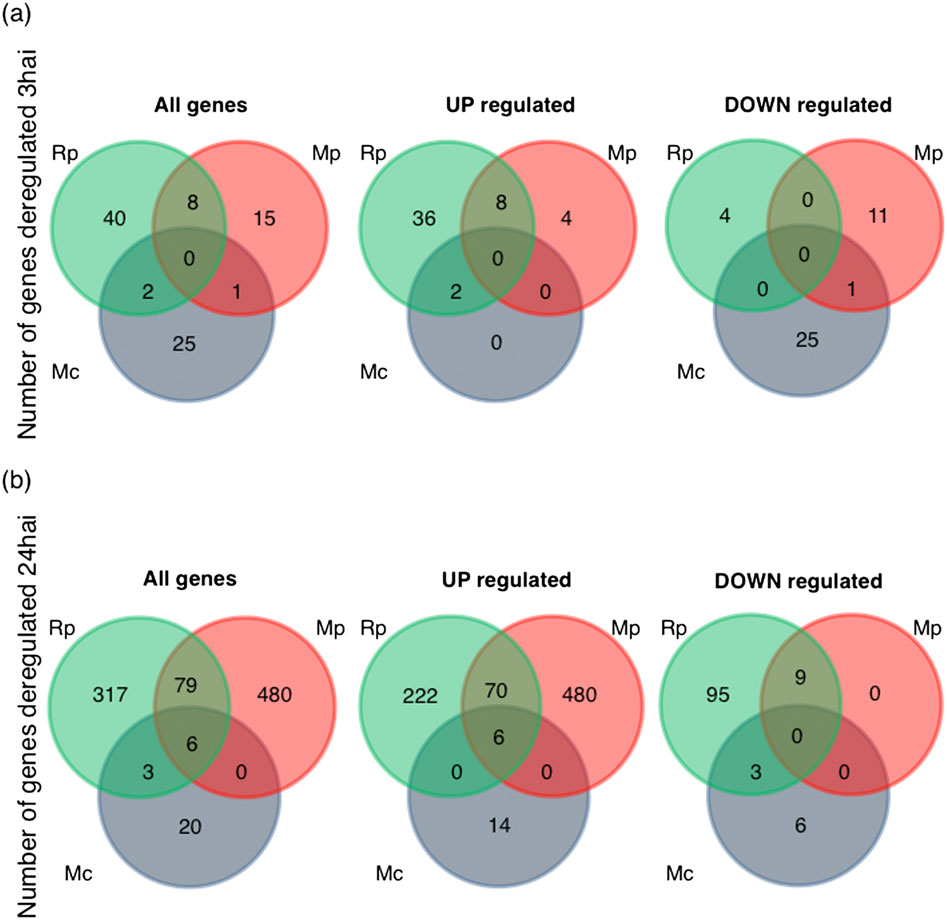
Venn diagrams showing the overlap between differentially expressed genes among different aphid interactions and timepoints. (a) Venn diagrams showing the numbers of genes differentially expressed from the no‐aphid control at 3 h after aphid challenge (paired *t*‐test, *P* ≤ 0.05). (b) Venn diagrams showing the numbers of genes differentially expressed from the control at 24 h after aphid challenge (paired *t*‐test, *P* ≤ 0.05). Rp, *Rhopalosiphum padi*; Mp, *Myzus persicae*; Mc, *Myzus cerasi*.

Overall analyses of the barley transcriptional responses during different aphid interactions suggested that some responses were more pronounced during specific aphid interactions or potentially aphid species specific. This led us to investigate potentially unique barley responses to each one of the aphid species. We performed pairwise analysis of the set of 974 genes to identify down‐regulated and up‐regulated genes per aphid species treatment per timepoint as compared with the no‐aphid control (Fig. 4 and Supporting Information Tables S4 and S5). The barley transcriptional response to aphids was more pronounced at the 24 h timepoint than at the 3 h timepoint, with 905 genes (24 h timepoint) versus 91 genes (3 h timepoint) being significantly differentially expressed in at least one of the aphid treatments compared with the non‐infested control (*P* ≤ 0.05).

For the 3 h timepoint, the barley transcriptional response was most pronounced upon interaction with R. *padi*, with 50 genes significantly differentially expressed. Forty of these were host interaction specific, with 36 being up‐regulated and four down‐regulated (Fig. 4a and Supporting Information Tables S4 and S5). GO annotation showed over‐representation of genes with predicted molecular functions in catalytic, transferase, hydrolase and chitinase activity (GO:0003824, GO:0016740, GO:0016798 and GO:0004568) in the set of 36 up‐regulated genes (Supporting Information Table S6). During the interaction with M. *persicae*, 24 genes were significantly differentially expressed, of which 15 were not affected by the other aphid species to a statistically significant level (Fig. 4a and Supporting Information Tables S4 and S5). Upon interaction with M. *cerasi*, a total of 26 genes were down‐regulated, of which only one was similarly affected by one of the other aphid interactions. Eleven barley genes were similarly affected by different aphid interactions at the 3 h timepoint, of which eight were shared between the R. *padi* and M. *persicae* interactions (Fig. 4a and Supporting Information Table S7).

At 24 h after aphid challenge, we observed a much stronger barley transcriptional response, especially in the case of the interactions with R. *padi* and M. *persicae* (Fig. 4b and Supporting Information Tables S4 and S5). The response to M. *cerasi* was weak, with just 20 genes differentially expressed (Fig. 4b and Supporting Information Tables S4 and S5). Therefore, for further detailed analyses, we specifically focused on the barley interactions with R. *padi* and M. *persicae* as detailed in the succeeding texts.

At the 24 h timepoint, 76 genes were significantly up‐regulated upon interaction with R. *padi* and M. *persicae*, whereas only nine genes were commonly down‐regulated between these interactions (Fig. 4b and Supporting Information Table S7). GO enrichment analyses showed that catalytic activity and oxidoreductase activity were over‐presented molecular functions for the up‐regulated set of genes (Supporting Information Table S8).

Out of the 317 genes significantly affected only upon R. *padi* interaction, 222 were up‐regulated (Fig. 4b and Supporting Information Table S4). GO enrichment showed an over‐representation of genes predicted to be involved in metabolic processes (GO:0008152, GO:0009987, GO:0044237 and GO:0044238), response to stimulus (GO:0050896) and response to stress (GO:0006950), and with over‐represented molecular functions in catalytic, transferase and kinase activity (GO:0003824, GO:0016740 and GO:0016301) (Supporting Information Table S6). In addition, BLAST similarity searches against rice and Arabidopsis databases (E < 10^−5^) revealed several genes predicted to function as WRKY transcription factors (WRKY3, 4, 31 and 50), cytochrome P450s, heat shock proteins and receptor‐like kinases. We then assessed whether this gene set showed a similar direction of regulation during the interactions with M. *persicae* by applying a log2 ratio = 1.0 cut‐off. This showed that actually 98% (218/222) of genes up‐regulated upon interaction with R. *padi* showed a similar direction of expression change upon M. *persicae* interaction (Supporting Information Table S4). For several of these genes, we even noted a higher fold change upon M. *persicae* interaction than upon R. *padi* interaction. Despite the higher fold changes, these genes were not found to be significantly up‐regulated upon M. *persicae* interaction, which is likely due to the overall stronger transcriptional response to this aphid species. Additionally, the response to R. *padi* included the specific down‐regulation of 95 genes, with no significant GO terms found (Fig. 4b and Supporting Information Tables S5 and S6). Of these 95 genes, only 11 were affected in a similar direction upon interaction with M. *persicae* based on a log2 ratio = 1.0 cut‐off. This suggests that the majority of these genes are specifically down‐regulated upon interaction with R. *padi*.

All of the 480 barley genes significantly affected specifically upon interaction with M. *persicae* were up‐regulated (Fig. 4b and Supporting Information Table S4). GO annotation revealed an over‐representation of genes with predicted functions in catalytic activity (GO:0003824) and copper binding (GO:0005507) (Supporting Information Table S6). BLAST similarity searches (E < 10^−5^) revealed many genes predicted to function as thionins, peroxidases, lipoxygenases, receptor‐like kinases and protein kinases (Supporting Information Table S4). To determine whether the 480 genes were regulated in a similar direction upon interaction with R. *padi*, we applied again a log2 ratio = 1.0 cut‐off. Only 14% (67/480) of the genes showed a similar trend in expression during R. *padi* interaction, although not to a statistically significant level (Supporting Information Table S4). Based on this, we conclude that the barley transcriptional response to M. *persicae* is stronger than the response to R. *padi*, with over 400 genes specifically up‐regulated during the M. *persicae* interaction.

MapMan analyses further confirmed the differences in barley transcriptional responses to R. *padi* and M. *persicae* after 24 h (Supporting Information Fig. S4). We observed the differences in functional categories between the two barley–aphid interactions, especially in the case of metabolic enzymes and stress‐related genes.

### Validation of barley transcriptional responses to aphids for selected genes across different barley cultivars

Based on the microarray data analyses, we selected 11 significant differentially expressed genes across different types of aphid interactions for validation by qRT‐PCR analyses. Three of the 11 genes, predicted to encode a cysteine proteinase (*MLOC* *_74627.1*), a Jasmonate ZIM‐domain transcription factor (*MLOC_9995.2*) and a WRKY4 transcription factor (*AK371133*), were similarly affected during interaction with R. *padi* and M. *persicae*. Eight of the 11 genes, predicted to encode three different thionins (*AK252675*, *AK359149* and *AK357884*), a late embryogenesis abundant protein 14 (*MLOC_5174.1*), a protein kinase (*AK373791*), a LOX2 (*AK357253.1*), a plant cadmium resistance protein 2‐like (*MLOC_79086.1*) kinase and a receptor‐like kinase (*MLOC_55207.1*), were significantly up‐regulated upon interaction with M. *persicae*. In addition to validating the microarray results for barley cultivar Optic, we also challenged cultivars Golden Promise and Morex with the different aphid species in three replicated experiments to determine if transcriptional responses were conserved across different barley cultivars.

**Figure 5:**
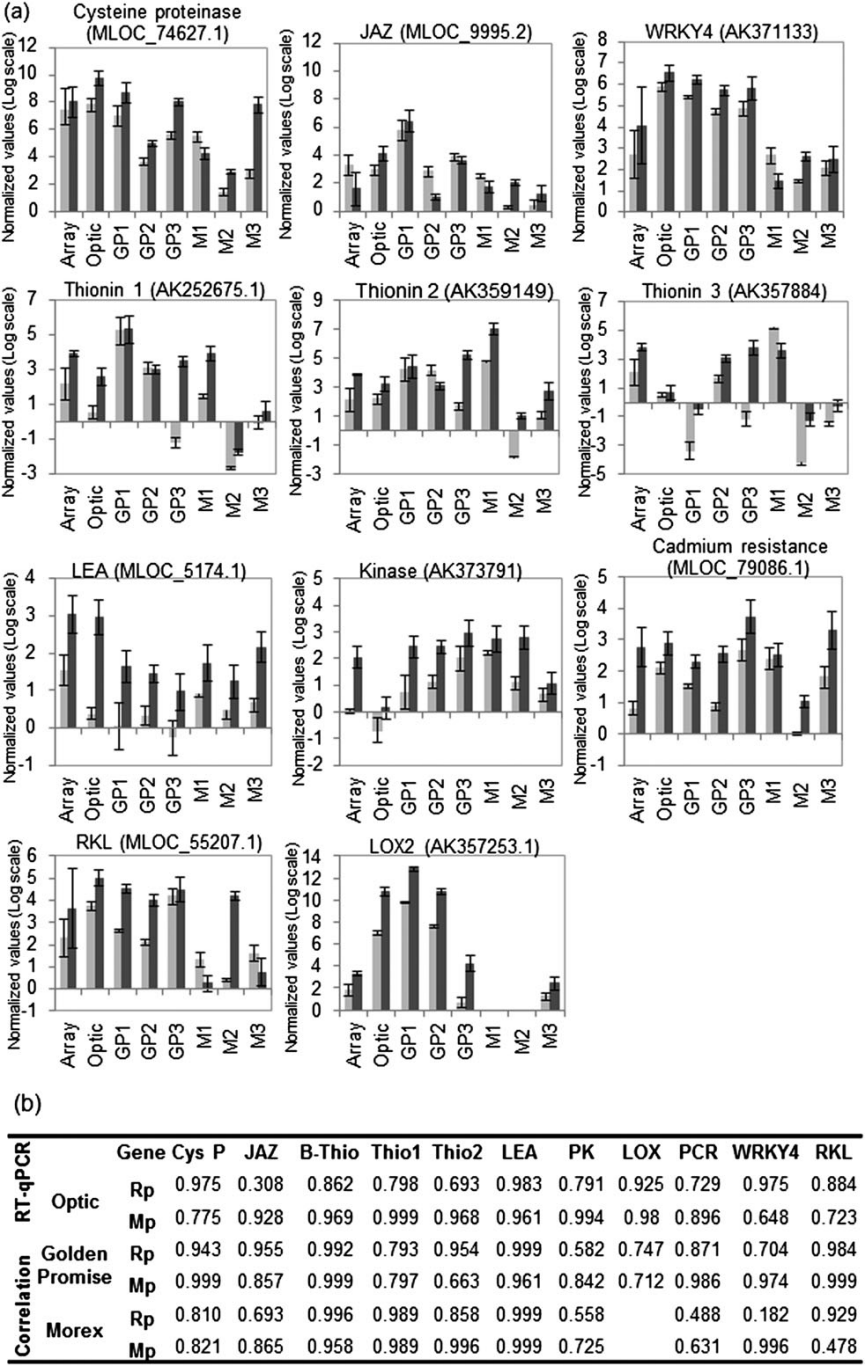
Validation of microarray data for selected genes using qRT‐PCR across different barley cultivars. Gene expression profiles of selected genes for validation of microarray results by qRT‐PCR. (a) The values represent the average of the log2 (ratio = E_sample_^Δct sample^/E_reference_^ΔCt reference^, where reference genes were *Actin 2*, *Ubiquitin* and *Pentatricopeptide*). Array indicates the average intensity of the three replicates according the microarray results. Optic indicates the expression level within the pool of three independent biological replicates used for microarray hybridization as determined by qRT‐PCR. GP1, GP2 and GP3 indicate the expression level in three independent biological replicates of barley cv. Golden Promise challenged with aphids. Morex 1, Morex 2 and Morex 3 indicate the expression level in three independent biological replicates of barley cv. Morex challenged with aphids. Light grey bars represent the log2 intensity during Rhopalosiphum padi (Rp) interaction, and dark grey bars represent the log2 intensity during Myzus persicae (Mp) interaction. Error bars indicate standard error. (b) Correlations between the expression values obtained in the microarray experiment and the values obtained by qRT‐PCR for the different barley cultivars (Optic, Golden Promise and Morex) upon interaction with Rp and Mp.

For 10 of the 11 selected barley genes, qRT‐PCR analyses confirmed the expression profile of the microarray data (cultivar Optic) (Fig. 5). For thionin 3, expression was outside reliable detection limits in the qRT‐PCR experiment, and as a result of this, we did not confirm the up‐regulation as observed in the microarray data (Fig. 5). When assessing expression profiles for the selected genes in cultivars Golden Promise and Morex, we noted that although the direction of expression was similar across cultivar–aphid interactions, the strength of response was slightly weaker in cultivar Morex for the majority of genes. In addition, we obtained variable expression profiles across replicates for the three selected thionins, but despite this observed up‐regulation in the majority of replicates, especially upon interaction with M. *persicae*. We also assessed the expression of thionins at a later timepoint (72 h) upon interaction with M. *persicae* and R. *padi* to determine if there was a more pronounced difference in expression at a later stage during the infestation process. Although thionins were up‐regulated upon infestation with both aphid species for 72 h, expression was higher for the M. *persicae* interaction than for the R. *padi* interaction (Supporting Information Fig. S5). As mentioned earlier, we identified 12 thionins significantly up‐regulated upon interaction with M. *persicae* in the microarray data, and further sequence analyses revealed that these genes are part of a large gene family in barley with over 39 members that show a high level of sequence similarity (Supporting Information Fig. S6). The primers and probes designed for qRT‐PCR analyses are unlikely to discriminate between the different members of this family (Supporting Information Fig. S7), which may be differently affected by aphid interaction, and this could explain the level of variation observed in this experiment. Overall, we were able to verify differential expression of selected barley genes upon aphid interaction across different barley cultivars and confirmed that some barley genes responded more strongly to M. *persicae* than to R. *padi*.

### Transient overexpression of barley thionins in *Nicotiana benthamiana* reduces *Myzus persicae* virulence

**Figure 6:**
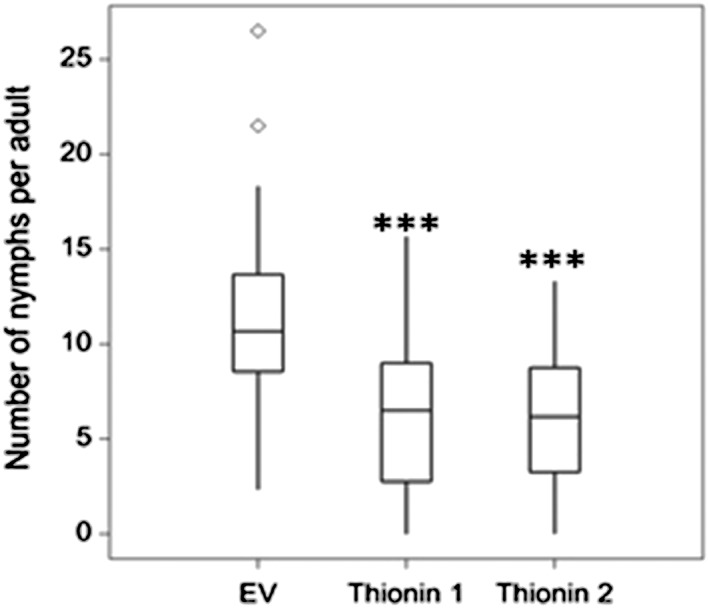
Ectopic expression of two barley thionin genes in *Nicotiana benthamiana* reduces Myzus persicae reproduction. Box plots showing the number of nymphs produced per adult aphid 14 d after infestation of leaf areas transiently expressing thionins or the vector control (EV). Differences between treatments were assessed by one‐way analysis of variance and *post hoc* Fisher's test (*P* ≤ 0.01). EV represents the empty vector, whereas thionin 1 (AK252675.1) and thionin 2 (AK359149) indicate the selected barley thionins.

Among the 480 genes significantly up‐regulated during the barley–M. *persicae* interaction, we identified 12 genes predicted to encode thionins, which are small proteins found specifically in plants that have antimicrobial activity (Bohlmann & Apel 1991; Thevissen *et al*. 1996). Based on this, we were interested to determine whether thionins impact aphid virulence. We cloned two of the barley thionin genes (AK252675 and AK359149) and ectopically expressed these under the 35S promoter by agroinfiltration in the solanaceous M. *persicae* host plant *N. benthamiana*. Infiltrated leaf areas were then challenged with three synchronized nymphs of M. *persicae*, and progeny per adult was counted over a 14 d period. Whilst aphids produced 11 nymphs per adult on leaf areas infiltrated with the *Agrobacterium* strain carrying the empty vector control, on the leaves transiently overexpressing the thionins progeny production was only six nymphs per adult, indicating a reduction of nearly 50% compared with the vector control (Fig. 6). To determine whether this observation was linked to possible defence activation due to ectopic expression of the thionins in *N. benthamiana*, we performed expression analyses of marker genes for salicylic acid signalling (PR‐1 and PAD4) and jasmonate signalling (PR4 and TP1). None of the markers were consistently differentially expressed upon transient ectopic expression of thionins (Supporting Information Fig. S8). Therefore, it is unlikely that activation of the corresponding defence signalling pathways is responsible for the observed reduction in reduced aphid performance.

## Discussion

In this study, we characterized the interaction of the monocot crop plant barley with three aphid species that differ in their ability to infest this plant species, to identify barley genes potentially involved in plant defences against aphids. This not only generated a comprehensive overview of how barley responds to aphid species during host, poor‐host and non‐host interactions at the transcriptional level but also revealed that thionins, which are highly up‐regulated upon aphid interaction, especially in the case of poor‐host interactions, may contribute to crop resistance.

Whilst we selected aphid species based on their ability to infest or not infest barley in a field environment, our experiments performed under controlled conditions showed that M. *persicae* was able to use barley as a host, but with poor performance, whereas M. *cerasi* was not even settling on this crop. Both M. *persicae* and M. *cerasi* are considered non‐pests of barley, and our findings suggest that the mechanisms underlying resistance against these two species are different. In the case of M. *cerasi*, it is possible that external plant cues, such as volatiles and epicuticular waxes, deter aphids from settling on barley and initiating probing (Powell *et al*. 2006). Alternatively, this species could be deterred by peripheral cues upon only one or few brief probes. Aphid probing, which allows aphids to contact the host cell cytoplasm as well as apoplast, is thought to be key in differentiation of host versus non‐host species (Powell *et al*. 2006). The frequent short probes by M. *persicae* and the low number of stylet pathways*,* in combination with poor growth of this aphid on barley, suggest that this aphid species is not able to feed optimally on this plant. Multiple short probes are also observed during incompatible interactions of other aphid species with both resistant host and non‐host plants. For example, frequent brief probes were observed for Macrosiphum euphorbiae feeding on resistant tomato plants containing the *Mi‐1* resistance gene (Kaloshian *et al*. 2000), indicating that although aphids were able to locate the phloem, their ability to successfully feed was impaired. Moreover, during non‐host interactions, frequent brief probes have been reported, which are sufficient to support transmission of non‐persistent viruses (Harrington *et al*. 1986; Schwarzkopf *et al*. 2013).

Potentially, the probing of the leaf tissue may cause damage that is responsible for the common activation of redox stress and jasmonate‐regulated genes as determined by GO analyses (Supporting Information Tables S2 and S3). The most highly up‐regulated gene for the R. *padi* and M. *persicae* interactions encoded a cysteine protease, with similarity to Arabidopsis *Senescence‐Associated Gene 12* (*SAG12*) (Supporting Information Table S7). Interestingly, infestation of Arabidopsis by M. *persicae* results in activation of *SAG* genes, including *SAG12*, and hypersenescence, potentially as part of the plant defence response (Pegadaraju *et al*. 2005). However, in barley, the function of this cysteine protease, and its potential link with senescence, has not yet been investigated.

A large number of genes were highly up‐regulated upon barley interaction with M. *persicae* compared with the interactions with the other aphid species, which could reflect specific activation of defences against this aphid species resulting in limited infestation success. One possibility is that the stronger barley transcriptional response to M. *persicae* is due to the frequent short probes observed. A number of genes predicted to function in cell wall‐related processes were specifically up‐regulated in response to M. *persicae*, which could be part of a damage response upon repeated probing of epidermal and mesophyll cell layers (Supporting Information Table S6). However, the combined number for the short probes and stylet pathways between the M. *persicae* and R. *padi* was quite similar among cultivars (Fig. 2b), suggesting that the frequency of total probes is comparable. Barley genes with more pronounced expression upon M. *persicae* interaction, as confirmed by qRT‐PCR analyses, were those encoding LOX2, a protein kinase, protein cadmium resistant 2, embryogenesis abundant protein 14, thionins and a receptor‐like kinase. Most of these genes are not well characterized in monocot crops. In Arabidopsis, we previously identified a *LEA* gene specifically activated during a non‐host interaction with aphids, but we found no evidence for activation of Arabidopsis thionins upon interaction with different aphid species (Jaouannet *et al*. 2015). Our experimental design took into account local barley responses (i.e. leaf areas within clip cages) to the different aphid species, and the impact on systemic responses remains to be investigated.

We identified several barley genes responsive to aphid infestation that show similarity to RLKs and E3 ubiquitin ligases implicated in *PTI* in other plant species (Supporting Information Table S4). *PTI* is not well characterized in barley, but the activation of these genes by aphids may reflect activation of components of *PTI* signalling pathways, which will need to be investigated further.

Our transcriptomics approach allowed us to identify barley thionin genes that, upon transient ectopic expression, decreased the susceptibility of a solanaceous plant species to M. *persicae*. Thionins are small peptides of around 5 kDa that contain six to eight cysteines involved in disulfide bridge formation, which are present in endosperm and leaves of cereals and several other plant species (Bohlmann & Apel 1991). The leaf‐specific thionins in barley are encoded by a large gene family, spanning 50–100 members per haploid genome (Bohlmann *et al*. 1988), and can be detected in either the plant cell wall as well as inside plant cells (Reimann‐Philipp *et al*. 1989b). Because of high levels of sequence similarity between different members of this family, we were unable to assess gene expression of specific thionins in either the microarray or qRT‐PCR experiments. Although in the microarray experiments, we noted a consistent up‐regulation of thionins, especially upon interaction with M. *persicae*, we obtained rather varying results in the validation qRT‐PCR experiments. Possibly, differences in gene expression among different members of the thionin family detected by the qRT‐PCR primers and probes could explain the observed variation. The induction of thionin expression increased beyond the timepoint selected for sampling for the microarray experiment, upon exposure to both M. *persicae* and R. *padi*. This suggests that thionin expression is not suppressed by R. *padi* during interactions with barley host plants. However, we cannot rule out that this aphid species may be able to suppress thionin function at the post‐transcriptional level or that R. *padi* is not affected by barley thionins.

Interestingly, overexpression of thionins in various plant species implicated these peptides in plant defences against plant pathogenic fungi (Bohlmann & Apel 1991), bacteria (Hao *et al*. 2016; Iwai *et al*. 2002) and chewing insects (Charity *et al*. 2005). Although it is thought that their activity relies on forming pores in the cell membranes of pathogens (Pelegrini & Franco 2005), it was recently shown that an Arabidopsis cell wall thionin suppressed cell death triggered by a fungal fruit body lectin from *Fusarium graminearum* upon direct binding (Asano *et al*. 2013). What is more, it was demonstrated that thionins can inhibit bacterial protein synthesis (García‐Olmedo *et al*. 1983). Our results suggest that thionins may be able to contribute to defences against phloem‐feeding aphids, but the underlying mechanism remains to be elucidated. An important next step is to confirm whether these thionins contribute to defences against aphids in barley and whether such defences are active against different aphid species that differ in their ability to infest this crop plant.

By performing basic characterization of barley interactions with different aphid species that differ in their ability to infest, we have shown that there are most likely different types of defences that either deter aphids or impair virulence. Transcriptome analyses revealed that barley responded more strongly to M. *persicae* (poor‐host interaction) than to R. *padi* (host interaction) and identified sets of genes that were specifically activated upon interaction with M. *persicae*. From the transcriptome dataset, we identified two genes, encoding thionins, which may contribute to defences against the broad host range pest M. *persicae*. By characterizing the interaction of M. *persicae* with poor‐host plants such as barley, we can identify plant genes that contribute to defences against this aphid pest. With the lack of crop resistance against broad host range pests such as M. *persicae*, the utilization of genes from poor‐host or non‐host plant species that confer aphid resistance may be a promising alternative for crop improvement.

## Competing interests

The authors declare that they have no competing interests.

## Supporting information


**Supplementary Figure 1:** **Assessment of variation between 3 h and 24 h control samples in microarray analyses.** (a) Venn diagram showing the overlap between the gene set identified as differentially expressed between the 3 h and 24 h control barley samples (331 genes, shown in the green circle on the left) and the gene set identified as differentially expressed genes across barley‐aphid interactions (974 genes, shown in the red circle on the right). The statistical analyses used were paired t‐test between samples (*p* ≤ 0.05). (b) Clustering analysis of the 854 differentially expressed across different aphid interactions after excluding the 120 genes differentially expressed between the 3 h and 24 h control samples that overlapped with the set of 974 differentially expressed genes across aphid interactions. Blue color indicates low expression level and red color indicates high expression level. No‐aphid control (C), R. padi (Rp), M. persicae (Mp), and M. cerasi (Mc) treatments are indicated.
**Supplementary Figure 2: Aphid probing on different cultivars Optic and Morex.** Images showing aphid stylet short probes and pathways in barley cv Optic and Morex. Pictures were taken two days after aphid challenge and visualized by staining with acid fuchsine. Images were taken with a light microscope. The stylets short probes and pathways are indicated arrows. Scale bars are 20 μm.
**Supplementary Figure 3: Additional pictures displaying callose deposition two days after probing for the aphid species** ***R. padi*** **,** ***M. persicae*** **and** ***M. cerasi*** **.** The callose was visualized with aniline blue and confocal microscope (wavelength 516 nm). Six leaf samples were used per aphid species per replicate and the experiment was done in three independent biological replicates. White arrows point stylet probing paths. Scale bars are 20 μm.
**Supplementary Figure 4: MapMan analyses of genes differentially regulated during interactions with** ***R. padi*** **and** ***M. persicae*** **at the 24 h timepoint.** Genes were mapped using the WSR‐test (Benjamin and Hochberg correction, (*p* ≤ 0.05). (a) Number of genes mapped for the barley‐R. padi in different functional categories (BINs). Significant BINs: RNA (blue), Signalling (red) and Misc. enzyme families (green) are indicated. (b) Number of genes mapped for the barley‐M. persicae interaction in different functional categories. Significant BINs: Proteins (lilac), Misc. enzyme families (green), RNA (blue), Stress (orange) and Secondary metabolism (grey) are indicated. Main BINs are indicated in bold.
**Supplementary Figure 5: Expression of 2 selected barley thionin genes 24 h and 72 h after aphid infestation.** Graph shows relative fold changes in expression 24 h and 72 h after aphid infestation with Rhopalosiphum padi and Myzus persicae compared to the control (uninfected barley). Thionin 1 (AK252675.1) and thionin 2 (AK359149) expression was assessed by qRT‐PCR in three independent biological replicates. Error bars represent the standard error.
**Supplementary Figure 6: Crustal‐Wallis (C‐W) alignment of thionins and thionin‐like peptides found across the barley genome.** C‐W multiple alignment of thionins and thionin‐like sequences (cds) found throughout the barley genome. Dark lilac and lilac shades indicate identical and highly similarity nucleotides, respectively.
**Supplementary Figure 7: Crustal‐Wallis alignment of thionins and their amplicons after qRT‐PCR.** C‐W multiple alignment of thionins sequences (HC genes database) (cds) identified by microarray and the amplicons resulting from virtual probes and primers proposed by Probe finder software (http://lifescience.roche.com) for the validated thionins (AK252675.1, AK359149 and AK357884). Dark lilac and lilac shades indicate identical and highly similarity nucleotides, respectively.
**Supplementary Figure 8: Expression of defence marker genes upon transient overexpression of two different thionins in *Nicotiana benthamiana*.** Bar graph shows the relative expression of defence markers genes upon transient overexpression of thionin 1 (AK252675.1), thionin 2 (AK359149) and the empty vector (EV) in *N. benthamiana* plants. The marker genes used were a salicylic acid (SA) marker (Nb_PR1), the jasmonate (JA) markers (Nb_PR4 and Nb_TP1) and a defence marker of M. persicae infestation phytoalexin deficient (PAD4) (Nb_PAD4). The expression was measured 4 days after infiltration by qRT‐PCR. Three independent replicates were used in this experiment. Asterisks indicate significant differences between treatments (Paired T‐test, *P* < 0.05). Error bars represent the standard error.
**Supplementary Table 1: Genes differentially expressed 3 h and 24 h after aphid challenge.** Genes were selected based on pair‐wise comparisons of each aphid treatment versus the no‐aphid control (paired t‐test, *p* ≤ 0.05). Indicated are the probe array code, normalized expression value (Log2 fold‐change), brief description, top hit and descriptions in rice, HarvEST35, Arabidopsis, AHRD Blast‐Hit‐Accession, AHRD Interpro‐ID, AHRD‐Quality‐Code and HC_genes, chromosome (POPSEQ) and position on the chromosome (POPSEQ cM). Rp indicates R. padi, Mp indicates M. persicae and Mc indicates M. cerasi.
**Supplementary Table 2: Hierarchical cluster lists for the genes differentially expressed 3 h and 24 h after aphid challenge.** Clustering of the 974 differentially expressed genes identified two main clusters (A and B), with 4 sub‐clusters of interest within cluster A and 2 within cluster B (*p* ≤ 0.05). Indicated are the probe array code, normalized expression value (Log2 fold change), brief description, top hit and descriptions in rice, HarvEST35, Arabidopsis, AHRD Blast‐Hit‐Accession, AHRD Interpro‐ID, AHRD‐Quality‐Code and HC_genes, chromosome (POPSEQ) and position on the chromosome (POPSEQ cM).
**Supplementary Table 3: GO enrichment analysis of the barley genes in different clusters and sub‐clusters lists based on homology to Arabidopsis.** Gene ontology of genes within the different clusters and sub‐clusters as listed in Supplementary Table 2 calculated with Fisher Exact Test (*p* ≤ 0.05). Gene Ontology codes for Biological process, Molecular function, Compartments, the Munich Information Centre for Protein Sequences (MIPS) or Plant Metabolic Network (AraCyc pathways, v11.5) information are included in the first column. The second column indicates terms related to the GO code. Columns 3 to 5 correspond to the frequency of the term observed in our data set, the frequency of the term observed in the whole genome, and the p‐value. The list of genes associated with each GO term is given in the last column.
**Supplementary Table 4: Genes specifically up‐regulated for each barley‐aphid interaction.** Genes specifically up‐regulated per aphid treatment and timepoint (paired t‐test, *p* ≤ 0.05). Indicated are the probe array code, normalized expression value (Log2 fold‐change), brief description, top hit and descriptions in rice, HarvEST35, Arabidopsis, AHRD Blast‐Hit‐Accession, AHRD Interpro‐ID, AHRD‐Quality‐Code and HC_genes, chromosome (POPSEQ) and position on the chromosome (POPSEQ cM). Rp indicates R. padi, Mp indicates M. persicae and Mc indicates M. cerasi.
**Supplementary Table 5: Genes specifically down‐regulated for each barley‐aphid interaction.** Genes specifically down‐regulated per aphid treatment and timepoint (paired t‐test, *p* ≤ 0.05). Indicated are the probe array code, normalized expression value (Log2 fold‐change), brief description, top hit and descriptions in rice, HarvEST35, Arabidopsis, AHRD Blast‐Hit‐Accession, AHRD Interpro‐ID, AHRD‐Quality‐Code and HC_genes, chromosome (POPSEQ) and position on the chromosome (POPSEQ cM). Rp stands for R. padi, Mp indicates M. persicae and Mc indicates M. cerasi.
**Supplementary Table 6: GO enrichment analysis of gene sets de‐regulated during each barley‐aphid interactions.** Gene ontology of the genes differentially regulated during specific barley‐aphid interactions listed in Supplementary Tables 5 and 6). GO was calculated using Fisher Exact Test (*p* ≤ 0.05). Gene Ontology codes for Biological process, Molecular function, Compartments, the Munich Information Centre for Protein Sequences (MIPS) or Plant Metabolic Network (AraCyc pathways, v11.5) information are included in the first column. The second column indicates a term related to the GO code. Columns 3 to 5 correspond to the frequency of the term observed in our data set, the frequency of the term observed in the whole genome and the p‐value. The list of genes associated with each GO term is given in the last column. Rp indicates R. padi, Mp indicates M. persicae and Mc indicates M. cerasi.
**Supplementary Table 7: Genes similarly affected across barley‐aphid interactions.** List of genes similarly affected across two barley‐aphid interactions as determined by paired t‐tests (*p* ≤ 0.05). Indicated are the probe array code, normalized expression value (Log2 fold change), brief description, top hit and descriptions in rice, HarvEST35, Arabidopsis, AHRD Blast‐Hit‐Accession, AHRD Interpro‐ID, AHRD‐Quality‐Code and HC_genes, chromosome (POPSEQ) and position on the chromosome (POPSEQ cM). Rp indicates R. padi, Mp indicates M. persicae and Mc indicates M. cerasi.
**Supplementary Table 8: GO enrichment analysis for genes similarly affected across different plant‐aphid interactions.** Gene ontology analysis of genes listed in Supplementary Table 7. Significance of GO annotation was calculated using Fisher Exact Test (*p* ≤ 0.05). Gene Ontology codes for Biological process, Molecular function, Compartments, the Munich Information Centre for Protein Sequences (MIPS) or Plant Metabolic Network (AraCyc pathways, v11.5) information are included in the first column. The second column indicates a term related to the GO code. Columns 3‐5 correspond to the frequency of the term observed in our data set, the frequency of the term observed in the whole genome, and the p‐value. The list of genes associated with each GO term is given in the last column. Rp stands for R. padi, Mp indicates M. persicae and Mc indicates M. cerasi.
**Supplementary Table 9: List of genes mapped using MapMan into different BINs.** List of genes specifically regulated after aphid challenge mapped in the different BINs and sub‐BINs using MapMan. The table shows the name of the BIN, number of elements in each BIN, and p‐value of the mapping per treatment and timepoint. Genes significantly mapped using the WSR‐test (*p* ≤ 0.05) are highlighted in light grey. Rp indicates R. padi and Mp indicates M. persicae.
**Supplementary Table 10: Taq‐Man probes, primers, and primer efficiency.** Indicated are the HC_genes, gene name, Taq‐Man probe number, primers, primer efficiency, and gene status in the microarray. Rp indicates R. padi and Mp indicates M. persicae.
**Supplementary Table 11:** List of 331 differentially expressed transcripts between the 3 h and 24 h control samples.
