# Supplementary figures and images for: Barley transcriptome analyses upon interaction with different aphid species identify thionins contributing to resistance
Source: Plant Cell Environ. 2017 Jul 18;40(11):2628–43. doi: 10.1111/pce.12979 (PMC6084319; doi:10.1111/pce.12979)

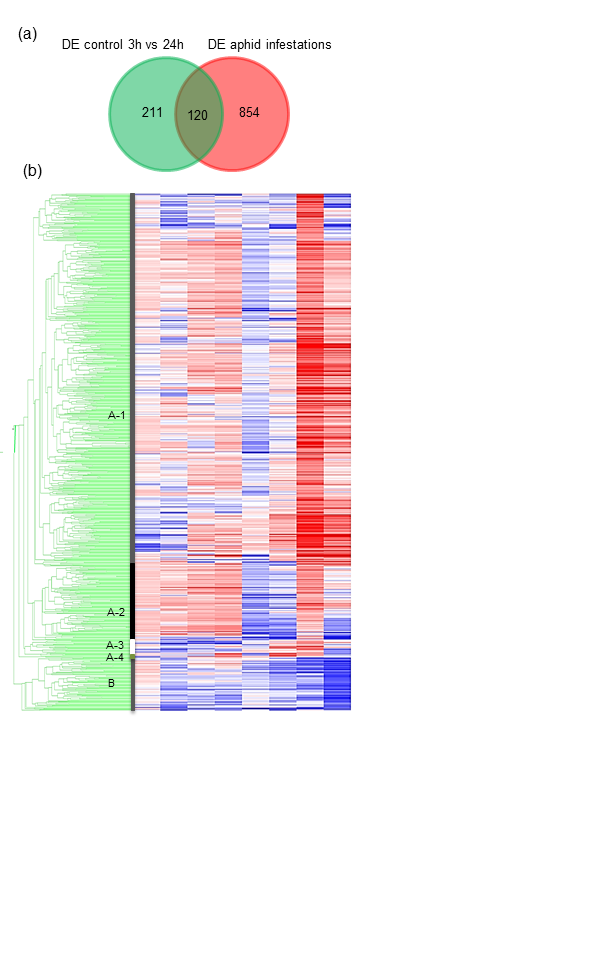

Supplement: Supplementary file 1 — Supplementary Figure 1: Assessment of variation between 3 h and 24 h control samples in microarray analyses. (a) Venn diagram showing the overlap between the gene set identified as differentially expressed between the 3 h and 24 h control barley samples (331 genes, shown in the green circle on the left) and the gene set identified as differentially expressed genes across barley‐aphid interactions (974 genes, shown in the red circle on the right). The statistical analyses used were paired t‐test between samples (p ≤ 0.05). (b) Clustering analysis of the 854 differentially expressed across different aphid interactions after excluding the 120 genes differentially expressed between the 3 h and 24 h control samples that overlapped with the set of 974 differentially expressed genes across aphid interactions. Blue color indicates low expression level and red color indicates high expression level. No‐aphid control (C), R. padi (Rp), M. persicae (Mp), and M. cerasi (Mc) treatments are indicated. Supplementary Figure 2: Aphid probing on different cultivars Optic and Morex. Images showing aphid stylet short probes and pathways in barley cv Optic and Morex. Pictures were taken two days after aphid challenge and visualized by staining with acid fuchsine. Images were taken with a light microscope. The stylets short probes and pathways are indicated arrows. Scale bars are 20 μm. Supplementary Figure 3: Additional pictures displaying callose deposition two days after probing for the aphid species R. padi , M. persicae and M. cerasi . The callose was visualized with aniline blue and confocal microscope (wavelength 516 nm). Six leaf samples were used per aphid species per replicate and the experiment was done in three independent biological replicates. White arrows point stylet probing paths. Scale bars are 20 μm. Supplementary Figure 4: MapMan analyses of genes differentially regulated during interactions with R. padi and M. persicae at the 24 h timepoint. Genes were mapped using th [file PCE-40-2628-s001.zip › SFig1.TIF]

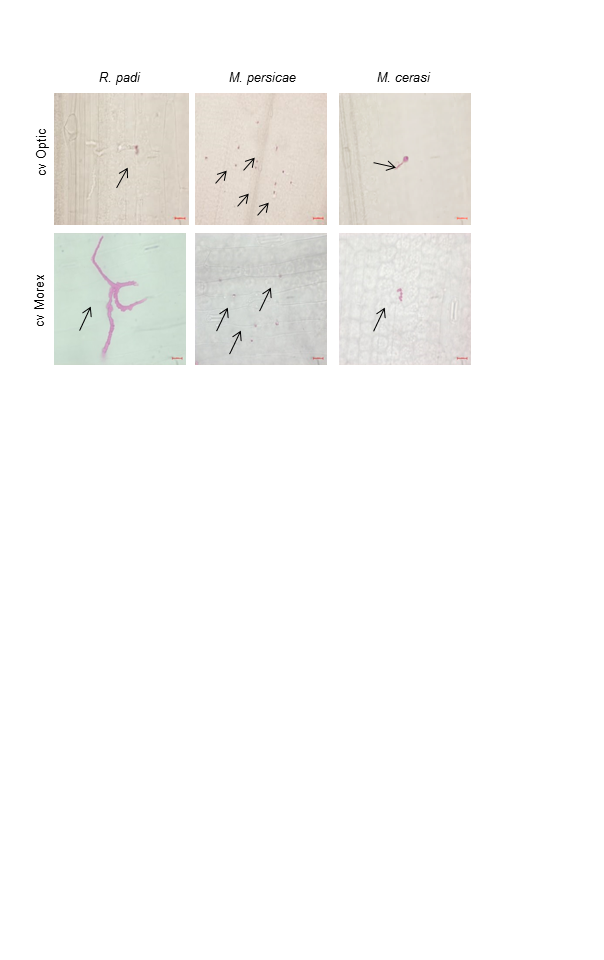

Supplement: Supplementary file 1 — Supplementary Figure 1: Assessment of variation between 3 h and 24 h control samples in microarray analyses. (a) Venn diagram showing the overlap between the gene set identified as differentially expressed between the 3 h and 24 h control barley samples (331 genes, shown in the green circle on the left) and the gene set identified as differentially expressed genes across barley‐aphid interactions (974 genes, shown in the red circle on the right). The statistical analyses used were paired t‐test between samples (p ≤ 0.05). (b) Clustering analysis of the 854 differentially expressed across different aphid interactions after excluding the 120 genes differentially expressed between the 3 h and 24 h control samples that overlapped with the set of 974 differentially expressed genes across aphid interactions. Blue color indicates low expression level and red color indicates high expression level. No‐aphid control (C), R. padi (Rp), M. persicae (Mp), and M. cerasi (Mc) treatments are indicated. Supplementary Figure 2: Aphid probing on different cultivars Optic and Morex. Images showing aphid stylet short probes and pathways in barley cv Optic and Morex. Pictures were taken two days after aphid challenge and visualized by staining with acid fuchsine. Images were taken with a light microscope. The stylets short probes and pathways are indicated arrows. Scale bars are 20 μm. Supplementary Figure 3: Additional pictures displaying callose deposition two days after probing for the aphid species R. padi , M. persicae and M. cerasi . The callose was visualized with aniline blue and confocal microscope (wavelength 516 nm). Six leaf samples were used per aphid species per replicate and the experiment was done in three independent biological replicates. White arrows point stylet probing paths. Scale bars are 20 μm. Supplementary Figure 4: MapMan analyses of genes differentially regulated during interactions with R. padi and M. persicae at the 24 h timepoint. Genes were mapped using th [file PCE-40-2628-s001.zip › SFig2.TIF]

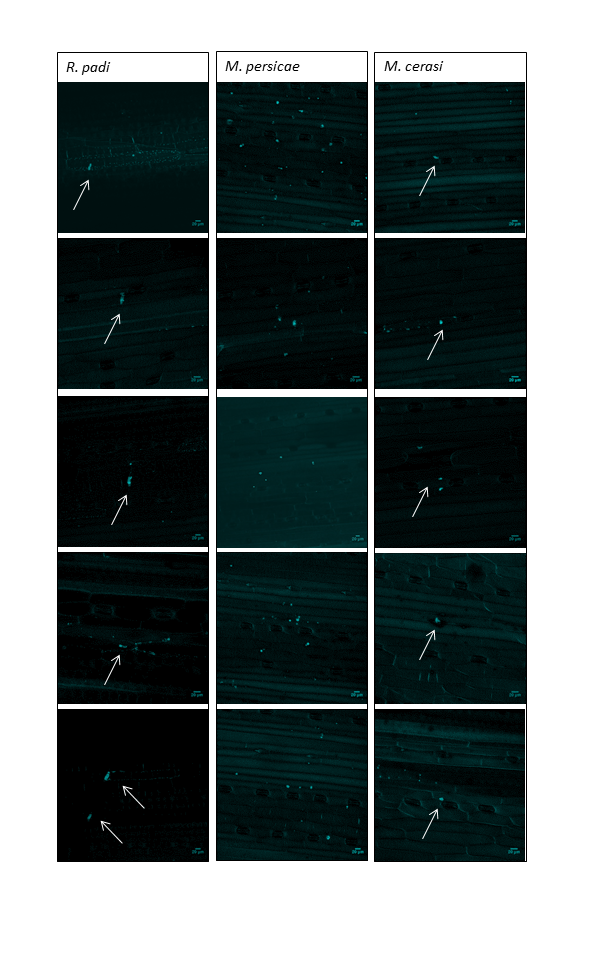

Supplement: Supplementary file 1 — Supplementary Figure 1: Assessment of variation between 3 h and 24 h control samples in microarray analyses. (a) Venn diagram showing the overlap between the gene set identified as differentially expressed between the 3 h and 24 h control barley samples (331 genes, shown in the green circle on the left) and the gene set identified as differentially expressed genes across barley‐aphid interactions (974 genes, shown in the red circle on the right). The statistical analyses used were paired t‐test between samples (p ≤ 0.05). (b) Clustering analysis of the 854 differentially expressed across different aphid interactions after excluding the 120 genes differentially expressed between the 3 h and 24 h control samples that overlapped with the set of 974 differentially expressed genes across aphid interactions. Blue color indicates low expression level and red color indicates high expression level. No‐aphid control (C), R. padi (Rp), M. persicae (Mp), and M. cerasi (Mc) treatments are indicated. Supplementary Figure 2: Aphid probing on different cultivars Optic and Morex. Images showing aphid stylet short probes and pathways in barley cv Optic and Morex. Pictures were taken two days after aphid challenge and visualized by staining with acid fuchsine. Images were taken with a light microscope. The stylets short probes and pathways are indicated arrows. Scale bars are 20 μm. Supplementary Figure 3: Additional pictures displaying callose deposition two days after probing for the aphid species R. padi , M. persicae and M. cerasi . The callose was visualized with aniline blue and confocal microscope (wavelength 516 nm). Six leaf samples were used per aphid species per replicate and the experiment was done in three independent biological replicates. White arrows point stylet probing paths. Scale bars are 20 μm. Supplementary Figure 4: MapMan analyses of genes differentially regulated during interactions with R. padi and M. persicae at the 24 h timepoint. Genes were mapped using th [file PCE-40-2628-s001.zip › SFig3.TIF]

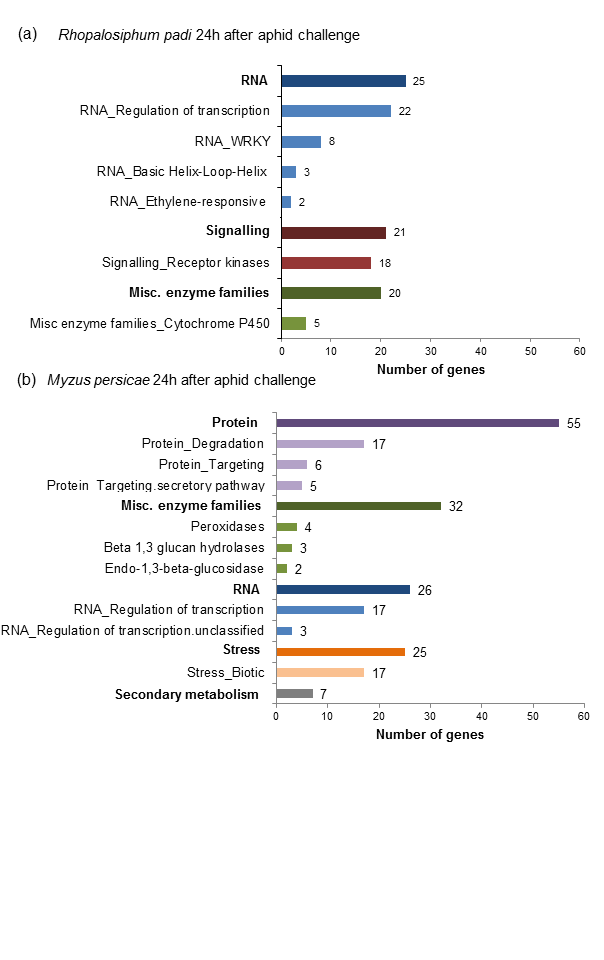

Supplement: Supplementary file 1 — Supplementary Figure 1: Assessment of variation between 3 h and 24 h control samples in microarray analyses. (a) Venn diagram showing the overlap between the gene set identified as differentially expressed between the 3 h and 24 h control barley samples (331 genes, shown in the green circle on the left) and the gene set identified as differentially expressed genes across barley‐aphid interactions (974 genes, shown in the red circle on the right). The statistical analyses used were paired t‐test between samples (p ≤ 0.05). (b) Clustering analysis of the 854 differentially expressed across different aphid interactions after excluding the 120 genes differentially expressed between the 3 h and 24 h control samples that overlapped with the set of 974 differentially expressed genes across aphid interactions. Blue color indicates low expression level and red color indicates high expression level. No‐aphid control (C), R. padi (Rp), M. persicae (Mp), and M. cerasi (Mc) treatments are indicated. Supplementary Figure 2: Aphid probing on different cultivars Optic and Morex. Images showing aphid stylet short probes and pathways in barley cv Optic and Morex. Pictures were taken two days after aphid challenge and visualized by staining with acid fuchsine. Images were taken with a light microscope. The stylets short probes and pathways are indicated arrows. Scale bars are 20 μm. Supplementary Figure 3: Additional pictures displaying callose deposition two days after probing for the aphid species R. padi , M. persicae and M. cerasi . The callose was visualized with aniline blue and confocal microscope (wavelength 516 nm). Six leaf samples were used per aphid species per replicate and the experiment was done in three independent biological replicates. White arrows point stylet probing paths. Scale bars are 20 μm. Supplementary Figure 4: MapMan analyses of genes differentially regulated during interactions with R. padi and M. persicae at the 24 h timepoint. Genes were mapped using th [file PCE-40-2628-s001.zip › SFig4.TIF]

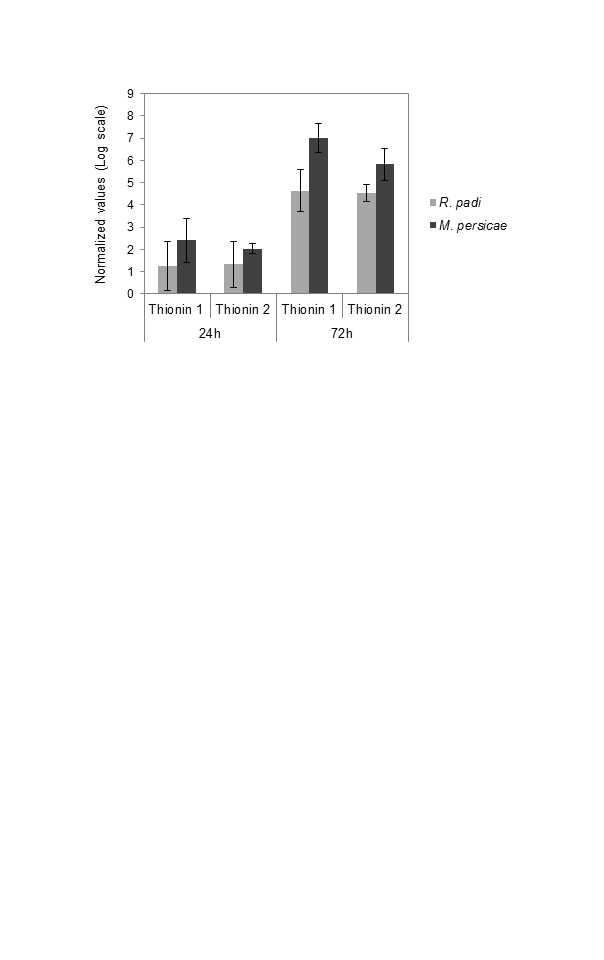

Supplement: Supplementary file 1 — Supplementary Figure 1: Assessment of variation between 3 h and 24 h control samples in microarray analyses. (a) Venn diagram showing the overlap between the gene set identified as differentially expressed between the 3 h and 24 h control barley samples (331 genes, shown in the green circle on the left) and the gene set identified as differentially expressed genes across barley‐aphid interactions (974 genes, shown in the red circle on the right). The statistical analyses used were paired t‐test between samples (p ≤ 0.05). (b) Clustering analysis of the 854 differentially expressed across different aphid interactions after excluding the 120 genes differentially expressed between the 3 h and 24 h control samples that overlapped with the set of 974 differentially expressed genes across aphid interactions. Blue color indicates low expression level and red color indicates high expression level. No‐aphid control (C), R. padi (Rp), M. persicae (Mp), and M. cerasi (Mc) treatments are indicated. Supplementary Figure 2: Aphid probing on different cultivars Optic and Morex. Images showing aphid stylet short probes and pathways in barley cv Optic and Morex. Pictures were taken two days after aphid challenge and visualized by staining with acid fuchsine. Images were taken with a light microscope. The stylets short probes and pathways are indicated arrows. Scale bars are 20 μm. Supplementary Figure 3: Additional pictures displaying callose deposition two days after probing for the aphid species R. padi , M. persicae and M. cerasi . The callose was visualized with aniline blue and confocal microscope (wavelength 516 nm). Six leaf samples were used per aphid species per replicate and the experiment was done in three independent biological replicates. White arrows point stylet probing paths. Scale bars are 20 μm. Supplementary Figure 4: MapMan analyses of genes differentially regulated during interactions with R. padi and M. persicae at the 24 h timepoint. Genes were mapped using th [file PCE-40-2628-s001.zip › SFig5.TIF]

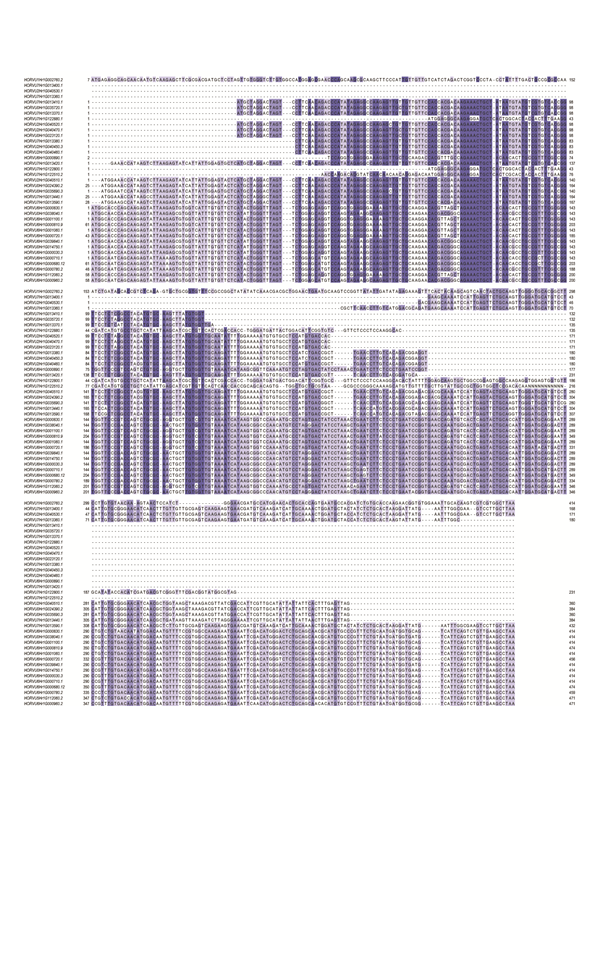

Supplement: Supplementary file 1 — Supplementary Figure 1: Assessment of variation between 3 h and 24 h control samples in microarray analyses. (a) Venn diagram showing the overlap between the gene set identified as differentially expressed between the 3 h and 24 h control barley samples (331 genes, shown in the green circle on the left) and the gene set identified as differentially expressed genes across barley‐aphid interactions (974 genes, shown in the red circle on the right). The statistical analyses used were paired t‐test between samples (p ≤ 0.05). (b) Clustering analysis of the 854 differentially expressed across different aphid interactions after excluding the 120 genes differentially expressed between the 3 h and 24 h control samples that overlapped with the set of 974 differentially expressed genes across aphid interactions. Blue color indicates low expression level and red color indicates high expression level. No‐aphid control (C), R. padi (Rp), M. persicae (Mp), and M. cerasi (Mc) treatments are indicated. Supplementary Figure 2: Aphid probing on different cultivars Optic and Morex. Images showing aphid stylet short probes and pathways in barley cv Optic and Morex. Pictures were taken two days after aphid challenge and visualized by staining with acid fuchsine. Images were taken with a light microscope. The stylets short probes and pathways are indicated arrows. Scale bars are 20 μm. Supplementary Figure 3: Additional pictures displaying callose deposition two days after probing for the aphid species R. padi , M. persicae and M. cerasi . The callose was visualized with aniline blue and confocal microscope (wavelength 516 nm). Six leaf samples were used per aphid species per replicate and the experiment was done in three independent biological replicates. White arrows point stylet probing paths. Scale bars are 20 μm. Supplementary Figure 4: MapMan analyses of genes differentially regulated during interactions with R. padi and M. persicae at the 24 h timepoint. Genes were mapped using th [file PCE-40-2628-s001.zip › SFig6.TIF]

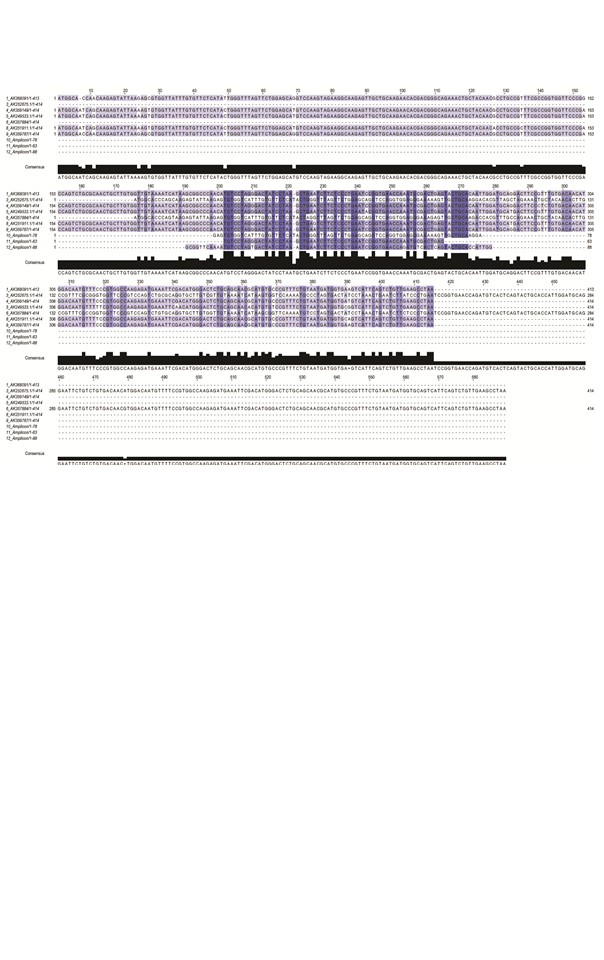

Supplement: Supplementary file 1 — Supplementary Figure 1: Assessment of variation between 3 h and 24 h control samples in microarray analyses. (a) Venn diagram showing the overlap between the gene set identified as differentially expressed between the 3 h and 24 h control barley samples (331 genes, shown in the green circle on the left) and the gene set identified as differentially expressed genes across barley‐aphid interactions (974 genes, shown in the red circle on the right). The statistical analyses used were paired t‐test between samples (p ≤ 0.05). (b) Clustering analysis of the 854 differentially expressed across different aphid interactions after excluding the 120 genes differentially expressed between the 3 h and 24 h control samples that overlapped with the set of 974 differentially expressed genes across aphid interactions. Blue color indicates low expression level and red color indicates high expression level. No‐aphid control (C), R. padi (Rp), M. persicae (Mp), and M. cerasi (Mc) treatments are indicated. Supplementary Figure 2: Aphid probing on different cultivars Optic and Morex. Images showing aphid stylet short probes and pathways in barley cv Optic and Morex. Pictures were taken two days after aphid challenge and visualized by staining with acid fuchsine. Images were taken with a light microscope. The stylets short probes and pathways are indicated arrows. Scale bars are 20 μm. Supplementary Figure 3: Additional pictures displaying callose deposition two days after probing for the aphid species R. padi , M. persicae and M. cerasi . The callose was visualized with aniline blue and confocal microscope (wavelength 516 nm). Six leaf samples were used per aphid species per replicate and the experiment was done in three independent biological replicates. White arrows point stylet probing paths. Scale bars are 20 μm. Supplementary Figure 4: MapMan analyses of genes differentially regulated during interactions with R. padi and M. persicae at the 24 h timepoint. Genes were mapped using th [file PCE-40-2628-s001.zip › SFig7.TIF]

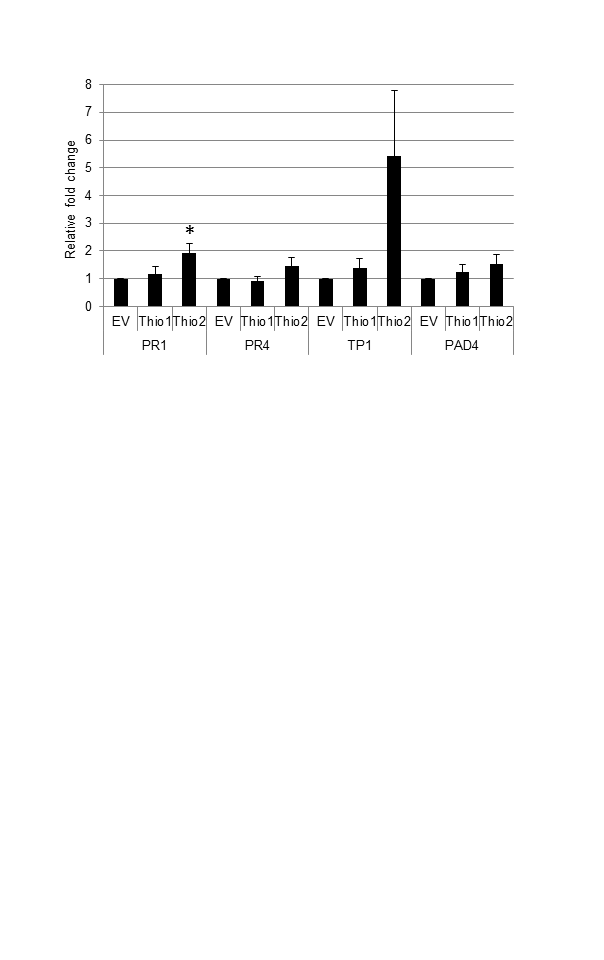

Supplement: Supplementary file 1 — Supplementary Figure 1: Assessment of variation between 3 h and 24 h control samples in microarray analyses. (a) Venn diagram showing the overlap between the gene set identified as differentially expressed between the 3 h and 24 h control barley samples (331 genes, shown in the green circle on the left) and the gene set identified as differentially expressed genes across barley‐aphid interactions (974 genes, shown in the red circle on the right). The statistical analyses used were paired t‐test between samples (p ≤ 0.05). (b) Clustering analysis of the 854 differentially expressed across different aphid interactions after excluding the 120 genes differentially expressed between the 3 h and 24 h control samples that overlapped with the set of 974 differentially expressed genes across aphid interactions. Blue color indicates low expression level and red color indicates high expression level. No‐aphid control (C), R. padi (Rp), M. persicae (Mp), and M. cerasi (Mc) treatments are indicated. Supplementary Figure 2: Aphid probing on different cultivars Optic and Morex. Images showing aphid stylet short probes and pathways in barley cv Optic and Morex. Pictures were taken two days after aphid challenge and visualized by staining with acid fuchsine. Images were taken with a light microscope. The stylets short probes and pathways are indicated arrows. Scale bars are 20 μm. Supplementary Figure 3: Additional pictures displaying callose deposition two days after probing for the aphid species R. padi , M. persicae and M. cerasi . The callose was visualized with aniline blue and confocal microscope (wavelength 516 nm). Six leaf samples were used per aphid species per replicate and the experiment was done in three independent biological replicates. White arrows point stylet probing paths. Scale bars are 20 μm. Supplementary Figure 4: MapMan analyses of genes differentially regulated during interactions with R. padi and M. persicae at the 24 h timepoint. Genes were mapped using th [file PCE-40-2628-s001.zip › SFig8.TIF]
